# Current Status of Artemisinin-Resistant *falciparum* Malaria in South Asia: A Randomized Controlled Artesunate Monotherapy Trial in Bangladesh

**DOI:** 10.1371/journal.pone.0052236

**Published:** 2012-12-18

**Authors:** Peter Starzengruber, Paul Swoboda, Hans-Peter Fuehrer, Wasif A. Khan, Verena Hofecker, Anja Siedl, Markus Fally, Oliver Graf, Paktiya Teja-Isavadharm, Rashidul Haque, Pascal Ringwald, Harald Noedl

**Affiliations:** 1 Institute of Specific Prophylaxis and Tropical Medicine, Medical University of Vienna, Vienna, Austria; 2 MARIB, Malaria Research Initiative Bandarban, Bandarban, Bangladesh; 3 International Centre for Diarrhoeal Disease Research, Dhaka, Bangladesh; 4 Department of Immunology and Medicine, United States Army Medical Component-Armed Forces Research Institute of Medical Sciences, Bangkok, Thailand; 5 World Health Organization, Global Malaria Programme, Geneva, Switzerland; Kenya Medical Research Institute – Wellcome Trust Research Programme, Kenya

## Abstract

**Objective:**

Recent reports indicate that first cases of genuine artemisinin resistance have already emerged along the Thai-Cambodian border. The main objective of this trial was to track the potential emergence of artemisinin resistance in Bangladesh, which in terms of drug resistance forms a gateway to the Indian subcontinent.

**Methods:**

We conducted an open-label, randomized, controlled 42-day clinical trial in Southeastern Bangladesh to investigate the potential spread of clinical artemisinin resistance from Southeast Asia. A total of 126 uncomplicated falciparum malaria patients were randomized to one of 3 treatment arms (artesunate monotherapy with 2 or 4 mg/kg/day once daily or quinine plus doxycycline TID for 7 days). Only cases fulfilling a stringent set of criteria were considered as being artemisinin-resistant.

**Findings:**

The 28-day and 42-day cure rates in the artesunate monotherapy (2 and 4 mg/kg) and quinine/doxycyline arms were 97.8% (95% confidence interval, CI: 87.8–99.8%), 100% (95% CI: 91.1–100%), and 100% (95% CI: 83.4–100%), respectively. One case of re-infection was seen in the artesunate high dose arm, and a single case of recrudescence was observed in the low dose group on day 26. No differences in median parasite and fever clearance times were found between the 2 artesunate arms (29.8 h and 17.9 h vs. 29.5 h and 19.1 h). Not a single case fulfilled our criteria of artemisinin resistance. Parasite clearance times were considerably shorter and *ex vivo* results indicate significantly higher susceptibility (50% inhibitory concentration for dihydroartemisinin was 1.10 nM; 95% CI: 0.95–1.28 nM) to artemisinins as compared to SE-Asia.

**Conclusion:**

There is currently no indication that artemisinin resistance has reached Bangladesh. However, the fact that resistance has recently been reported from nearby Myanmar indicates an urgent need for close monitoring of artemisinin resistance in the region.

**Trial Registration:**

ClinicalTrials.gov NCT00639873.

## Introduction

Virtually all malaria-endemic countries have adjusted their treatment guidelines to spreading resistance to practically all traditional antimalarials and now recommend artemisinin-based combination therapies (ACTs) as first-line therapy for the treatment of uncomplicated falciparum malaria [1]. In Bangladesh ACTs have only very recently been introduced as official first line treatment. Artemisinins have never previously been used on any significant scale and baseline efficacy data for the individual combination partners used in ACTs in Bangladesh are sparse. First reports indicating reduced overall susceptibility and slow clinical response of *P. falciparum* to artemisinin derivates with individual cases of genuine resistance to artesunate have been reported from the Thai-Cambodian and more recently the Thai-Myanmar border suggesting a westward spread of resistance [2]–[5]. Several studies published in recent years indicate that the efficacy of ACTs remains high in Bangladesh [6]–[8]. However, the relative impact of resistance to artemisinins, their partner drugs and other factors contributing to clinical treatment response remains uncertain. The spread of resistance to artemisinin derivates, currently the most essential drugs for the treatment of falciparum malaria, could very well result in one of the most devastating events in the history of malaria control in the 21^st^ century and potentially endanger all recently initiated malaria elimination efforts. Historical evidence from traditional antimalarials suggests that drug resistance has a tendency to spread westwards from its origins in Southeast Asia [9]–[11]. The central question of this trial was therefore whether artemisinin resistance has already reached the eastern borders of Bangladesh, which in terms of drug resistance traditionally forms a gateway to the Indian subcontinent.

This study was part of the Artemisinin Resistance Confirmation, Characterization and Containment Project (ARC3), an effort coordinated by the World Health Organization (WHO) and funded by the Bill & Melinda Gates Foundation, to define the problem, extent, and spread of artemisinin resistance in South and Southeast Asia. The protocol for this trial and supporting CONSORT checklist are available as supporting information; see Checklist S1 and Protocol S1. Registration information: Clinicaltrials.gov Identifier NCT00639873.

## Methods

### Ethics statement

Written informed consent was obtained from all study participants or their legal representatives, and the study was approved by the ethical review boards of the Medical University of Vienna, the Research Ethics Review Committee of the World Health Organization and the Ethical Review Committee of the International Centre for Diarrhoeal Disease Research, Bangladesh (ICDDR, B).

### Study site and participants

This study was conducted at the MARIB (Malaria Research Initiative Bandarban) field site in Bandarban in Southeastern Bangladesh. Study participants were male or nonpregnant female patients aged 8 to 65 years, who presented with acute symptomatic *Plasmodium falciparum* mono-infections with a parasite density of 1,000–100,000 asexual parasites/μL as determinate by microscopy on a screening slide. All female patients between the age of 12 and 65 were required to have a negative human chorionic gonadotropin (hCG) urine pregnancy test (Clear Dip, megro, Wesel, Germany). All females of childbearing potential were required to use an acceptable contraception throughout the duration of their participation in the study. Patients who presented with one of the following criteria were excluded from study participation: sings or symptoms of severe malaria (as defined by WHO criteria) [12], mixed malaria infection on the screening slide, a previous history of intolerance or hypersensitivity to the study drugs or to drugs with similar chemical structures, malaria drug therapy in the past 30 days by history, symptoms of severe vomiting (no food or inability to take food during the previous 8 hours).

### Study design

The study was a single-center, open label, randomized, controlled clinical trial specifically designed to detect the potential emergence of artemisinin resistance. Patients were hospitalized for 7 days (the duration of study drug administration), or until complete fever and parasite clearance, whichever took longer. Participants were asked to return for follow-up visits on days 14, 21, 28, 35, and 42 and whenever signs and symptoms consistent with malaria reappeared. Insecticide-treated mosquito nets were provided to all patients to limit the chances of reinfection after discharge from the hospital. Patients who failed initial therapy in the artesunate groups were treated with 7 days of oral quinine (10 mg/kg body weight, three times daily), patients in group 3 who failed initial therapy received 3 days Coartem according to the national treatment guidelines.

### Sample size

Enrollment target was 100 evaluable subjects (i.e. study participants reaching primary study endpoint). Subjects were enrolled in the 3 groups at a ratio of 2∶2∶1. Any dropouts (e.g. withdrawals, lost to follow-ups, etc.) were replaced by continuing randomization until a total number of 100 evaluable subjects were reached.

### Randomization

Subjects were allocated unique identification codes and were randomly assigned to one of the three treatment groups using block randomization in blocks of 10 by reference to a statistical series based on random sampling numbers drawn up for each patient by the study staff; the details of the series were unknown to any of the investigators or to the coordinator and were contained in a set of sealed envelopes.

### Study drugs

Artesunate (Artesunate 50 mg tablets, Guilin Pharmaceutical Co., Ltd., Batch no. 071201) was provided by WHO, Geneva, Switzerland, quinine sulphate BP (Jasoquin® 300 mg tablets, Batch no. 189) was purchased from Jayson Pharmaceuticals Ldt., Dhaka, Bangladesh and doxycyline hyclate (Doxycap® 50 mg tablets, Batch no. 75853 and Doxycap® 100 mg tablets, Renata Limited, Dhaka, Bangladesh, Batch no. 858–001) was procured from local suppliers.

Patients in all 3 treatment groups received oral antimalarial therapy for 7 days. Artesunate monotherapy was administrated over 7 days either as 2 mg/kg body weight once daily in the low dose arm or as 4 mg/kg body weight once daily in the higher dose arm. Patients in the control group received oral quinine (10 mg/kg body weight 3 times daily) plus doxycyline (2 mg/kg twice daily) over 7 days. A study physician observed study drug administration and the full dose was repeated if vomiting occurred within one hour after drug administration. Concomitant medication was provided for the symptomatic treatment of fever, headache, myalgia, nausea and/or vomiting.

### Evaluation

After enrollment a full medical history, the results of the physical examination, vital signs, adverse events, clinical signs and symptoms as well as medication history were recorded daily until day 7 and whenever patients returned for follow-up. A urine pregnancy test was performed on admission day for all females between the age of 12 and 50. Venous blood samples for complete blood count and blood chemistry were collected using the Vacuette^®^ blood collection system (Greiner Bio-One GmbH, Kremsmünster, Austria) on day 0 and 3 or whenever clinically warranted. For DNA fingerprinting, RNA, and *in vitro* drug sensitivity assays blood samples were collected on day 0 and in case of recurrence of parasitemia. Plasma samples for determining drug levels were collected from all patients on days 0 and 6.

Peripheral malaria blood smears were performed from finger prick blood at admission and after this twice daily until two consecutive slides were confirmed to be negative as well as at every follow-up visit. Giemsa-stained thick and thin blood smears were examined by a microscopist who was blinded to treatment, clinical status of the study subject and to any other results. All slides that were positive on any follow-up day were reexamined by a second microscopist. In case of a difference in the result (positive/negative or species) between the two microscopists, the blood smear was re-examined by a third senior microscopist and this reading was accepted as final result. Parasite density was determined based on the count of parasites per 200 white blood cells (thick film) or 2000 red blood cells (thin film). A total of 200 immersions oil field were screened before a blood film was considered as negative as well as to rule out mixed infections.

### Efficacy measures

Outcome measures for the clinical study were based on the criteria set forth by the WHO [13]. The primary endpoint was adequate clinical and parasitological response (ACPR) on Day 28 and Day 42 (PCR-corrected). Treatment failure was divided into early treatment failure (ETF), late clinical failure (LCF), and late parasitological failure (LPF). Secondary efficacy variables were parasite and fever clearance time.

### Reinfections

Blood samples for DNA fingerprinting were collected before drug intake and in case of reemergence of parasites to distinguish between reinfection and recrudescence. Gene loci (GLURP, MSP1 and MSP2) of these samples were compared by polymerase chain reaction (PCR) [14].

### *Ex vivo* drug susceptibility

All fresh parasite samples obtained on day 0 and on the day of recrudescence were tested in a histidine-rich protein 2 (HRP2) drug susceptibility assay for their sensitivity to dihydroartemisinin (DHA), artesunate, and artemisinin. The cultures and enzyme-linked immunosorbent assays (ELISA) were carried out as previously described [15], [16]. The chloroquine-sensitive 3D7 *P. falciparum* clone was used as a reference and for quality control of drug-coated culture plates.

### Pharmacokinetics

Venous blood samples were collected from all patients in arm 1 and 2 into heparinized tubes just before and 60, 120, and 180 (±15 minutes) after artesunate intake on day 0 and day 6. The samples were centrifuged immediately and plasma stored at −20°C before transferring to −80°C within a maximum of 3 weeks. DHA levels were determined by LC-MS as described elsewhere [17]. Any subject in whom the mean of the areas under the curve on Days 0 and 6 lay below the overall mean −1 SD was considered to have inadequate drug levels for DHA.

### Defining criteria for artemisinin resistance

The definition of artemisinin resistance has been a controversial issue ever since the discovery of the first cases of resistance. We therefore used a conservative approach in defining resistance. Only cases fulfilling all of the following criteria were defined as being artemisinin-resistant: recrudescence during follow up or failing to clear parasites after 7 days of directly observed monotherapy with 2 or 4 mg/kg/day of artesunate in the absence of any signs of vomiting after drug administration or malabsorption and with an enrollment parasite density of <100,000 asexual parasites/µL; prolonged PCT indicating poor clinical response; exclusion of re-infection by PCR; pharmacokinetic parameters confirming adequate (greater than mean in cures -1SD) drug levels for DHA; *in vitro* drug sensitivity tests on clinical isolates suggesting elevated inhibitory concentrations for artemisinins and/or genetic makers indicating reduced drug susceptibility [18].

### Data analysis

Cure rates were estimated using Kaplan-Meier survival analysis to calculate the proportion of aparasitemic patients at each point in time. Nonlinear regression analysis based on a polynomial regression model was used to calculate individual *in vitro* drug inhibitory concentrations. Continuous data were compared by t-test/ANOVA. Data not showing normal distribution were compared by Mann-Whitney U/Kruskal-Wallis ANOVA. Proportions were compared using Fisher's Exact/Chi^2^ test.

## Results

### Study flow

**Figure 1:**
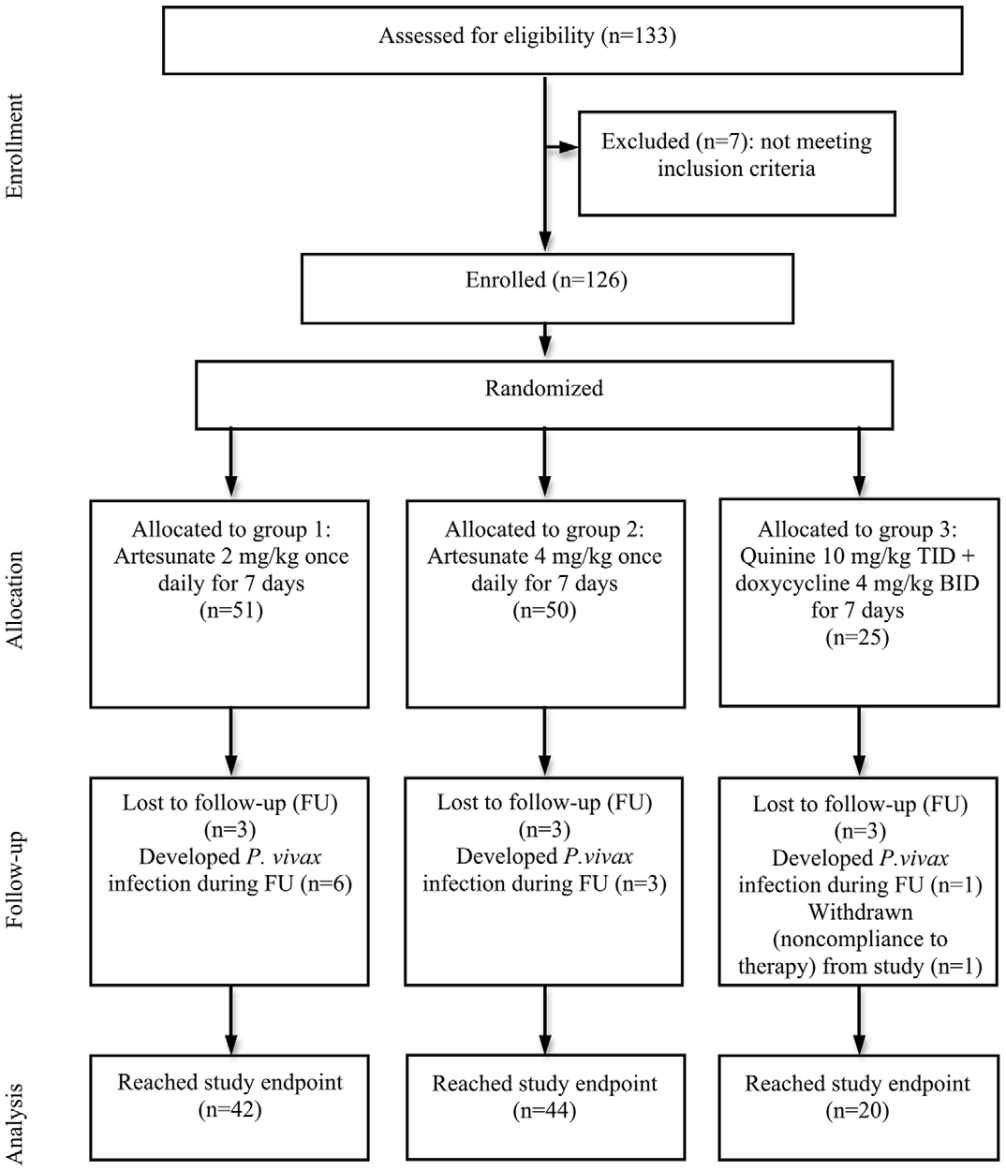
Flow diagram of the progress through the phase of the trial from enrollment until data analysis for the 3 groups.

A total of 133 patients were screened and 126 were randomized to one of the three treatment groups out of whom 106 reached primary study endpoint (Fig. 1). Nine patients were lost to follow up and one was withdrawn from the study. Another 10 subjects developed *P. vivax* infections between day 21 and 42 (6 in Arm 1, 3 in Arm 2, and 1 in Arm 3). All dropouts, except for the one who was withdrawn, occurred after completing the full treatment course and after clearing parasites and fever. Forty-two subjects were evaluable for primary study end point in Arm 1, 44 in Arm 2, and 20 in the control group. All three arms were well matched in regard to sex, age, weight, height, and parasite density on enrollment (Table 1).

**Table 1:** Demographic and baseline characteristics of study subjects.

		Arm	
Characteristics	1	2	3
No. of male/female patients (%)	41(32.5)/10 (7.9)	42 (33.3)/8 (6.3)	22 (17.5)/3 (2.4)
Age, mean years ± SD	24.3(12.5)	26.4(13.6)	23.0 (11.4)
Weight, mean kg ± SD	44.8 (11.8)	47.7 (12.1)	42.8 (10.6)
Height, mean cm ± SD	155.6 (15.2)	158.4 (15.1)	155.7 (12.8)
Temperature, mean °C	37.7 (1.2)	37.7 (1.3)	37.9 (1.2)
Enrollment parasite density, GM/µL	7910	8315	9137

**NOTE:** SD, standard deviation; °C, degree Celsius; GM/µL, geometric mean/micro Liter.

### Clinical efficacy

All patients successfully cleared their asexual parasitemia within the first week of treatment. The 28 and 42-day cure rate for Arm 1 by Kaplan-Meier analysis was 97.8% (95% confidence interval, CI: 87.8–99.8%), 100% (95% CI: 91.1–100%) for Arm 2, and 100% (95% CI: 83.4–100%) for Arm 3 (Table 2). Of the two treatment failures, one was classified as late clinical failure in Arm 1 on day 26 (recrudescence by PCR) and the second as late parasitological failure in Arm 2 on day 42 (reinfection by PCR). The patient classified as recrudescence on day 26 had a parasite clearance time of 21.7 h and a parasitemia of 8,666 parasites/µL. Not a single case fulfilled our criteria of artemisinin resistance.

**Table 2:** Primary (cure rates) and secondary clinical endpoints (parasite and fever clearance) for all treatment groups.

		Arm				
Characteristics	1	2	3	P value Arm1 vs Arm 2	P value Arm1+2 vs 3	P value Arm 1 vs Arm 2 vs Arm 3
Cure rate Day 28						
No. of patients evaluable on Day 28	45	46	22			
No. of cured patients	44	46	22	0.92	0.89	0.995
Cure rate % (95%CI)	97.8	100	100			
	(87.8–99.8)	(91.1–100)	(83.4–100)			
Treatment failure	1	0	0	1	1	0.596
Cure rate Day 42 (PCR-corrected)						
No. of patients evaluable for study end point Day 42	42	44	20			
No. of cured patients	41	43	20	0.89	0.92	1
Cure rate %(95%CI)	97.8	100	100			
	(87.8–99.8)	(91.1–100)	(83.4–100)			
Treatment failure	1	1	0	1	1	1
PCT, h						
Median (IQR)	29.8	29.5	43.1			
	(24.3–32.8)	(19.8–32.4)	(33.6–54.8)	0,327	<0.001	<0.001
PCT50						
Median (IQR)	9.3	8.5	29.5	0.542	0.002	0.006
	(6.5–18.4)	(7.1–17.5)	(7.1–31.0)			
PCT90						
Median (IQR)	17.6	17.4	30.0	0.905	<0.001	0.003
	(8.9–19.7)	(8.3–19.5)	(18.2–31.6)			
Parasite reduction ratio						
Median (IQR) after 12h	0.93	0.78	70.37	0.610	<0.001	<0.001
	(0.0–2.98)	(0.0–3.08)	(5.71–187.84)			
Median (IQR) after 24h	0.0	0.0	0.8	0.857	<0.001	<0.001
	(0.0–0.0)	(0.0–0.0)	(0.0–7.14)			
Median (IQR) after 36h	0.0	0.0	0.0	1	0.019	0.062
	(0.0–0.0)	(0.0–0.0)	(0.0–0.57)			
FCT, h						
Median (IQR)	17.9	19.1	29.3			
	(4.6–20.7)	(6.6–23.6)	(16.0–43.1)	0.263	0.001	0.003

**NOTE:** 95%CI, 95% confidence interval; PCT, parasite clearance time in hours; IQR, interquartile range; PCT50, time in hours to 50% clearance of parasite density; PCT90, time in hours to 90% clearance of parasite density.

The median parasite clearance times (PCTs) were similar in the artesunate lower and higher dose arms with 29.8 (interquartile range, IQR: 24.3–32.8) h and 29.5 (IQR: 19.8–32.4) h, respectively. With 43.1 (IQR: 33.6–54.8) h the PCT in the control group was significantly (P<0.001) slower. At the first measurement more than 24 hours after initiation of treatment 13.7% (95% CI: 6.15–26.9) of the patients in Arm 1, 16.0% (95% CI: 7.6–29.7) in Arm 2 and 70.8% (95% CI: 48.8–86.6) in the control group were still parasitemic. All patients in the artesunate arms had cleared their parasitemia after 48 hours, whereas 16,7% (95% CI: 5.5–38.2) in the control group were still positive. After 72 hours all patients tested negative for asexual malaria parasites. The median 50% parasite reduction times were 9.3 (IQR: 6.5–18.4), 8.5 (IQR: 7.1–17.5), and 29.5 (IQR: 7.1–31.0) h, for Arms 1–3, respectively. The corresponding mean 90% parasite reduction times were 17.6 (IQR: 8.9–19.7), 17.4 (IQR: 8.3–19.5), and 30.0 (IQR: 18.2–31.6) h.

The median parasite reduction ratios at 12, 24, 36, and 48 h (calculated as 100 minus the percentage reduction from the baseline level) were 0.93 (IQR: 0–2.98), and 0.0 (IQR: 0.0–0.0) in Arm1; 0.78 (IQR: 0.0–3.08), and 0.0 (IQR: 0–0–0.0) in Arm 2; 70.37 (IQR: 5.71–187.84), 0.8 (IQR: 0.0–7.14), 0.0 (IQR: 0.0–0.57) and 0.0 (0.0–0.0) in Arm 3. The ratios in the artesunate groups were lower than in the quinine group after 12 and 24 hours (P<0.001). There was no difference in the parasite reduction ratio between the artesunate subgroups.

With a median FCT of 17.9 (IQR: 4.6–20.7), 19.1 (IQR: 6.6–23.6), and 29.3 (IQR: 16.0–43.1) h, for Arms 1–3, respectively, the artesunate arms showed a significantly (P = 0.001) faster clinical response than the control group.

### *Ex vivo* findings

Out of a total of 106 parasite samples obtained on the day of study admission 83 (78.3%; 95% CI: 69.0–85.5) were successfully tested for their *in vitro* drug susceptibility to DHA, artemisinin and artesunate. The geometric mean 50% inhibitory concentration (IC_50_) for DHA was 1.10 nM (N = 83; 95% CI: 0.95–1.28 nM; range: 0.34–8.52), 1.37 nM (N = 83; 95% CI: 1.17–1.61; range: 0.48–12.18) for artesunate and 3.74 (N = 82; 95% CI: 3.15–4.43; range: 0.80–27.42) nM for artemisinin, respectively. No correlation was observed between PCT and IC_50_s of DHA, artemisinin or artesunate in the artesunate arms. The IC_50_ of DHA, artesunate and artemisinin for the reference clone 3D7 was 0.78 nM (95% CI: 0.71–0.85), 1.6 nM (95% CI: 1.32–1.94) and 5.27 nM (95% CI: 4.57–6.08), respectively. The patient classified as recrudescence had an IC_50_ of 1.28, 1.74 and 5.53 nM for DHA, artesunate and artemisinin, respectively.

### Pharmacokinetics

Plasma DHA levels measured after drug intake of the first dose and last dose were used to determine whether there had been adequate drug exposure in patients treated with artesunate. The total mean drug exposure estimated by the area under curve (±SD) of DHA in the low dose group after the first and last dose was 1,365±665 ng/mLxh and 592±228 ng/mLxh, and in the high dose group 2,936±1,576 ng/mLxh and 1,463±736 ng/mLxh, respectively. Adequate DHA concentrations were defined as the mean ±1 SD. The area under the curve estimated for the recrudescent patient in the low dose group was considered adequate at 1,384 ng/mLxh after the first and 1,077 ng/mLxh last dose, respectively.

### Adverse Events

No serious adverse events were observed. All treatment regimes were generally well tolerated. Out of 78 recorded adverse events, 48 (61.5%; 95% CI: 49.8–72.1%) were classified as drug-related. No difference in the proportion of patients with/without adverse events was observed between the three groups (P = 0.91). The most common adverse event in all 3 arms was gastrointestinal disorder.

## Discussion

First indications of high failure rates associated with the extensive use of ACTs in Thailand were reported in a paper published in 2006 suggesting the possibility of poor clinical response to ACTs along the Thai-Cambodian border and coincide with the first reports of the experimental induction of *in vitro* artemisinin resistance [19], [20]. Recent studies have shown that the first cases of genuine artemisinin resistance have already emerged in the region [2]–[5] raising the question of how far artemisinin resistance has already spread.

This study using a systematic *in vivo*-*ex vivo* approach was conducted in Southeastern Bangladesh in close proximity to the borders of India and Myanmar and was specifically designed to answer the question of whether clinical artemisinin resistance has already spread to the region. As compared to Southeast Asia the major difference in terms of exposure of malaria parasites to artemisinins is the fact that in Bangladesh ACTs have only been introduced very recently and that the local parasite populations have therefore not been exposed to artemisinins on any significant scale. The parasite phenotype seen in Bangladesh is therefore likely to be representative of Asian *P. falciparum* populations before the introduction of artemisinins. Historical data suggest that in terms of the spread of resistance from Southeast Asia the malaria-endemic regions of Bangladesh may represent an important gateway to the Indian Subcontinent.

Our data clearly indicate that in this region there is currently no evidence for spreading or *de novo* emerging artemisinin resistance. Although a single case of recrudescence was observed, this case did not fulfill our stringent criteria for artemisinin resistance [18]. Even 7 days of artesunate monotherapy with a total dose much higher than the one commonly used in artemisinin-based combinations typically result in a certain proportion of failures. The one patient who developed a recrudescence received 700 mg total dose (2.04 mg/kg) as compared to a typical artesunate dose of 300 mg in artemisinin-based combinations. All other parameters, however, indicate that this case was a drug failure rather than a failure due to drug resistance. As compared to the Thai-Cambodian border PCTs were considerably shorter (28.2 vs. 57.6 in a recently published study from Cambodia; P<0.001) [3]. Our *ex vivo* data confirm earlier published data suggesting a continuous and significant decrease in *ex vivo* artemisinin susceptibility from Bangladesh throughout western to eastern Thailand and Cambodia [4]. There was also no increase in the IC_50_ for artemisinins as compared to our earlier studies in the same region suggesting the absence of a temporal trend towards reduced sensitivity [6], [21].

The discussion of the role and importance of poor clinical response and failures with 7 days of high-dose artemisinins along the Thai-Cambodian border has largely been focusing on the question of whether innately resistant clones occur naturally as a fixed subset of parasite populations from different locales. Our data from an area in Asia where artemisinins have as yet not been deployed on any significant scale would suggest that this is not the case.

In its early stages reduced drug susceptibility is not necessarily reflected in an increased proportion of treatment failures. Clinical response parameters (particularly parasite clearance) and intrinsic *ex vivo* drug sensitivity therefore play a crucial role in detecting early stages of compromised drug sensitivity [18]. In this study all indicators seem to suggest as yet uncompromised drug sensitivity to artemisinins in southeastern Bangladesh. The comparison of *ex vivo* drug sensitivity between Bangladesh and Cambodia seems to closely match the major differences in treatment response [2]–[5].

The obvious advantage of artemisinins is their capacity to quickly reduce the initial parasite burden making them highly successful even in combination treatment courses as short as 3 days (as typically used in ACTs). However, with parasite clearance times reaching 100 hours or more in selected patients in Cambodia [2], [3] artemisinins cannot fulfill their main function.

Both, the prolongation in parasite clearance and persisting parasitemia 72 hours after initiation of treatment are strong indicators for reduced susceptibility or artemisinin resistance [22]. In our study neither were PCTs prolonged nor did a single patient have detectable parasites after 72 hours. Our findings are an obvious contrast to recent findings in western Thailand and Cambodia, where the positivity rate at 72 h was as high as 21.9% and where PCTs were almost twice as high as in our study with individual times reaching values close to the traditional classification of RII resistance [2], [3], [23].

In summary, there is currently no indication of compromised drug sensitivity to artemisinin derivatives in the region. The rapid reduction in parasite densities as well as the high efficacy seen with 7 days artesunate monotherapy in Bangladesh suggests that the phenomenon seen in Cambodia and along the eastern borders of Myanmar is likely to be based on the selection of parasite populations with reduced sensitivity under drug pressure rather than due to innately resistant clones occurring naturally as a fixed subset of parasite populations. So far artemisinins have not been used on any significant scale in Bangladesh but the situation is likely to change in the future, either due to resistance spreading from Southeast Asia or due to de novo emergence of resistance under drug pressure. These data therefore also provide an important baseline for future assessments of temporal and geospatial trends in artemisinin resistance in the region.

## Supporting Information

Checklist S1 **CONSORT Checklist.**
(DOC)

Protocol S1 **Trial Protocol.**
(PDF)
