# Supplementary material for: Current Status of Artemisinin-Resistant falciparum Malaria in South Asia: A Randomized Controlled Artesunate Monotherapy Trial in Bangladesh
Source: PLoS One. 2012 Dec 18;7(12):e52236. doi: 10.1371/journal.pone.0052236 (PMC3525560; doi:10.1371/journal.pone.0052236)
Supplement: Protocol S1 — Trial Protocol. (PDF) [file pone.0052236.s002.pdf]

ARTEMISININ RESISTANCE IN BANGLADESH

Protocol ID Number:
MUW # 83/2008
ICDDR,B # 2008-008

Principal Investigators:
Dr. Harald Noedl
Dr. Wasif Ali Khan

Version 1.2

Version Number: *Version 1.2*

17 April 2008

SIGNATURE PAGE

The signatures below document the approval of this protocol and the attachments, and provide the necessary assurances that this study will be conducted according to all stipulations of the protocol, including all statements regarding confidentiality and according to local legal and regulatory requirements.

Investigators:

Signed: ______________________________ Date: ______________

Name: Dr Harald Noedl

Signed: ______________________________ Date: ______________

Name: Dr Wasif Ali Khan

Table of Contents

page
Statement of Compliance 1
Signature Page 1
List of Abbreviations 5
Protocol Summary 7

1 Key Roles 9
2 Background Information and Scientific Rationale 11
  2.1 Background Information 11
Artesunate for the treatment of malaria 13
Artesunate: Efficacy and Safety Profile 15
  Artesunate Safety Data 16
  2.2 Rationale 19
  2.3 Potential Risks and Benefits 20
    2.3.1 Potential Risks 20
    2.3.2 Potential Benefits 20
3 Objectives 22
4 Study Design 23
5 Study Population 25
  5.1 Selection of the Study Population 25
  5.2 Study Site 25
  5.3 Inclusion Criteria 25
  5.4 Exclusion Criteria 26
  5.5 Randomization Procedures 26
6 Study Procedures/Evaluations 27
  6.1 Study Procedures 27
  6.2 Laboratory Evaluations 27
    6.2.1 Laboratory Evaluations/Assays 28
    6.2.2 Special Assays or Procedures 28
    6.2.3 Specimen Collection, Preparation, Handling and Shipping 28
7 Study Schedule 30
  7.1 Screening 30
  7.2 Follow-up and Final Visits 30
  7.3 Criteria for Discontinuation or Withdrawal of a Subject 31
8 Assessment of Outcome Measures 32
  8.1 Specification of the Appropriate Outcome Measures 32
    8.1.1 Primary Outcome Measures (Clinical Outcomes) 32
    8.1.2 Primary Outcome Measures (Laboratory Outcomes) 32
    8.1.3 Secondary Outcome Measures 33
9 Safety assessment and reporting 34
  9.1 Definition of Adverse Event (AE) 34
  9.2 Definition of Serious Adverse Event (SAE) 34
  9.3 Safety Reporting Procedures 34
    9.3.1 Serious Adverse Event Detection and Reporting 36

Table of Contents - *continued*

page
9.3.2 Type and Duration of the Follow-up of Subjects After Adverse Events...... 36
9.4 Halting Rules 37
10 Statistical Considerations 38
10.1 Study Outcome Measures and Analysis 38
10.2 Sample Size Considerations 38
10.3 Participant Enrollment and Follow-Up 39
11 Access to Source Data/Documents 40
12 Ethics/Protection of Human Subjects 41
12.1 Declaration of Helsinki 41
12.2 Institutional Review Board 41
12.3 Informed Consent 41
12.3.1 Informed Consent Process 42
12.4 Subject Confidentiality 42
12.5 Future Use of Stored Specimens 43
13 Literature References 44

SUPPLEMENTS/APPENDICES
A: Study Schedule
B: Definitions

ABBREVIATIONS

ACPR	Adequate Clinical and Parasitological Response
ACT	Artemisinin-based Combination Therapy
AE	Adverse Event
AS	Artesunate
AV	Atrioventricular
CFR	Code of Federal Regulations
CIOMS	Council for International Organizations of Medical Sciences
CRF	Case Report Form
CRO	Clinical Research Organization
CRR	Continuing Review Report
DHA	Dihydroartemisinin
DHSP	Division of Human Subjects Protection
DNA	Deoxyribonucleic Acid
DOT	Directly Observed Therapy
EKG	Electrocardiogram
ETF	Early Treatment Failure
FCT	Fever Clearance Time
FDA	Food and Drug Administration
FWA	Federal-Wide Assurance
GCP	Good Clinical Practice
GCT	Gametocyte Clearance Time
GLP	Good Laboratory Practice
GMP	Good Manufacturing Practice
hCG	Human Chorionic Gonadotropin
HIV	Human Immunodeficiency Virus
HRP2	Histidine-rich Protein II
HRPO	Human Research Protection Office
HSRRB	Human Subjects Research Review Board
ICDDR,B	International Centre for Diarrhoeal Research, Bangladesh
IC	Inhibitory Concentration
ICF	Informed Consent Form
ICH	International Conference on Harmonization
IEC	Independent or Institutional Ethics Committee
IRB	Institutional Review Board
ISM	Independent Safety Monitor
IV	Intravenous
LCF	Late Clinical Failure
LPF	Late Parasitological Failure
LTF	Late Treatment Failure

M5AS	Mefloquine 1250 mg plus Artesunate
MARIB	Malaria Research Initiative Bandarban
MEF	Mefloquine
MOP	Manual of Procedures
mRNA	Messenger Ribonucleic Acid
MUW	Medical University of Vienna
N	Number (typically refers to subjects)
NIH	National Institutes of Health
ORP	Office of Research Protection
P.	Plasmodium
PCR	Polymerase chain reaction
PCT	Parasite Clearance Time
Pf.	Plasmodium falciparum
PK	Pharmacokinetic
PI	Principal Investigator
QC	Quality Control
QT	Q-T Interval
RBC	Red Blood Cell
RNA	Ribonucleic Acid
SAE	Serious Adverse Event
SMC	Safety Monitoring Committee
SOP	Standard Operating Procedure
SSP	Study Specific Procedure
WBC	White Blood Cell
WHO	World Health Organization

Protocol Summary

Title: Artemisinin Resistance in Bangladesh

Protocol Identifier: ICDDR,B # 2008-008 MUW # 83/2008

Funding: World Health Organization

Rationale: Recent data indicate that the first cases of artemisinin resistance have emerged in Asia. A reduced overall sensitivity of *P. falciparum* to artemisinin derivatives both *in vivo* as well as *in vitro* has been demonstrated along the Cambodian-Thai border. Once it starts spreading, resistance to artemisinin derivatives, currently the most essential antimalarial drugs for the treatment of *Plasmodium falciparum* malaria, could very well be the most devastating event in the history of malaria control in the 21st century. There is therefore an urgent need for early detection of emerging artemisinin resistance, particularly in Asia, where artemisinins have been used for a long time. This study is part of an effort coordinated by the World Health Organization (WHO) to define the problem, extent, and spread of artemisinin resistance in South and Southeast Asia and includes 3 countries: Cambodia, Thailand, Bangladesh. The aim of this study is to assess baseline data for artemisinin sensitivity and efficacy in Bangladesh, a country where artemisinins have never been used to any significant extent.

Objectives: PRIMARY:

- To determine the baseline efficacy of artesunate monotherapy for the treatment of uncomplicated falciparum malaria in Bangladesh as well as to define the impact of varying doses of artesunate on treatment outcome.

SECONDARY:

- Validate treatment response parameters (PCT, FCT) for their role in predicting failures.
- To evaluate the current malaria *in vitro* drug sensitivity situation in this area.
- To validate potential genetic markers of artemisinin resistance.

Study Design: Randomized, open label clinical trial

Population: Up to 134 patients may be enrolled to achieve 100 evaluable subjects randomized in 3 groups (2 artesunate monotherapy arms and one control arm, ratio 2:2:1). Otherwise healthy *P. falciparum* infected malaria patients aged 8 to 65 years.

Study Location: Bandarban District, Southeastern Bangladesh

Study Site: Bandarban Sadar Hospital, Bangladesh

Study Drugs: Artesunate monotherapy versus quinine and doxycycline for 7 days.

Treatment Group	Sample Size	Artesunate		Quinine		Doxycycline	
		Dosing mg/kg/day	Total Dose/kg	Dosing mg/kg/day	Total Dose/kg	Dosing mg/kg/day	Total Dose/kg
1 (Artesunate)	40	2	14	N/A	N/A	N/A	N/A
2 (Artesunate)	40	4	28	N/A	N/A	N/A	N/A
3 (Controls)	20	N/A	N/A	30	210	4	28

Study Duration: Approximately 18 months from enrollment of the first subject. The study may be repeated the following year after amendment of the protocol. Such an amendment will have to be reviewed and approved by all reviewing IRBs.

Subject Duration: Duration per subject is 42 days

Endpoints: Primary clinical outcome is cure (Adequate Clinical and Parasitological Response – ACPR as defined by WHO criteria) on Day 28 and 42. Secondary outcome measures are time until parasite, fever, and gametocyte clearance (PCT, FCT, and GCT).

Abstract: A total number of up to 134 patients may be enrolled to achieve 100 evaluable volunteers with acute uncomplicated falciparum malaria will be randomly assigned one of 3 arms to be treated with artesunate monotherapy or quinine and doxycycline for 7 days at a ratio of 2:2:1. The study design will be based on the WHO recommendations for the 'Assessment and Monitoring of Antimalarial Drug Efficacy for the Treatment of Uncomplicated Falciparum Malaria' (WHO, 2003). Study participants will be otherwise healthy malaria patients aged 8 to 65 years with uncomplicated falciparum malaria recruited at the Bandarban Sadar Hospital, Bangladesh.

The artesunate will be administered orally (a single dose of 2 or 4 mg/kg/day) over a total duration of 7 days by directly observed therapy.

Patients will be admitted to the hospital for the duration of study drug administration or until all signs and symptoms of malaria have disappeared, whichever comes later. Thereafter they will be followed as

outpatients until Day 42 with scheduled follow-up visits on Day 14, 28, 35, and 42.

In vitro drug sensitivity assays will be performed from samples on inclusion and in case of recrudescence. Drug levels will be measured on the first and last day of therapy.

Primary clinical outcome is cure (Adequate Clinical and Parasitological Response - ACPR) on Day 28 and 42. Secondary outcome measures are time until parasite, fever, and gametocyte clearance (PCT, FCT, and GCT). Parasite genotyping will be used to distinguish recrudescences from reinfections by PCR for patients in whom recrudescences cannot be fully excluded. Subjects will be monitored for clinical adverse events throughout the study duration.

Blood will be drawn on the day of admission (before initiating therapy) for *in vitro* drug sensitivity testing and for PCR (markers of drug resistance and to distinguish recrudescence from reinfection by genotyping). Malaria smears will be prepared twice daily until parasite clearance and on Days 7, 14, 21, 28, 35, and 42 or whenever symptoms consistent with malaria appear. Plasma samples for determining drug levels will be obtained on the first and last day of therapy. Study participation for each individual will be 42 days.

1 KEY ROLES

Individuals: **Principal Investigators:**

Harald Noedl, M.D., M.C.T.M., Ph.D.
Institute of Specific Prophylaxis
and Tropical Medicine,
Medical University of Vienna
Kinderspitalgasse 15
A-1090 Vienna, Austria
Tel.: +(43-1) 4277-64882
Fax.: +(43-1) 4277-64889
Email: harald.noedl@meduniwien.ac.at

Wasif Ali Khan M.B.B.S, M.H.S.
Clinical Sciences Division
ICDDR,B - Centre for Health and Population Research
Mohakhali, Dhaka-1212, Bangladesh
Tel.: +(880-2) 600171-78 Ext.: 2333
Fax.: +(880-2) 883116
Email: wakhan@icddrb.org

Associate Investigators:

Peter Starzengruber, M.D.
Institute of Specific Prophylaxis
and Tropical Medicine,
Medical University of Vienna
Kinderspitalgasse 15
A-1090 Vienna, Austria
Tel.: +(43-1) 4277-64882
Fax.: +43-1-4277-64889
Email: peter.starzengruber@meduniwien.ac.at

Rashidul Haque, M.B.B.S., Ph.D.
Laboratory Research Department
Laboratory Sciences Division
ICDDR,B - Centre for Health and Population Research
Mohakhali, Dhaka-1212, Bangladesh
Tel.: +(880-2) 600171-78 – Ext.: 2411
Fax.: +(880-2) 883116
Email: rhaque@icddrb.org

Ching Swe Phru, M.B.B.S
Medical Officer.
Sadar Hospital Bandarban.
Bandarban 4600, Bangladesh
Tel.: +88-0361 62544
Email: phrucs@gmail.com

Hans-Peter Fuehrer, M.Sc..
Institute of Specific Prophylaxis
and Tropical Medicine,
Medical University of Vienna
Kinderspitalgasse 15
A-1090 Vienna, Austria
Tel.: +(43-1) 4277-64882
Fax.: +43-1-4277-64889
Email: Hans-Peter@chello.at

Institutions:

Medical University of Vienna
A-1090 Vienna, Austria
Tel.: +(43-1) 4277-64882; Fax: +(43-1) 4277-64889

ICDDR,B - Centre for Health and Population Research
Mohakhali, Dhaka-1212, Bangladesh
Tel.: +(880-2) 600171-78; Fax: +(880-2) 883116

MARIB – Malaria Research Initiative Bandarban
A-1180 Vienna, Austria
Tel.: +43-699-81807989

Institutional Review Boards:

Ethical Review Committee of the ICDDR,B
Ethical Review Committee of the Medical University of Vienna
Research Ethics Review Committee of the World Health Organization

2 BACKGROUND INFORMATION AND SCIENTIFIC RATIONALE

2.1 Background Information

In Bangladesh artemisinin-based combination therapies (ACTs) have only recently been introduced as the official first line therapy for the treatment of uncomplicated falciparum malaria. However, currently chloroquine remains the most commonly used drug for the treatment of uncomplicated falciparum malaria. There are no baseline data on the clinical efficacy of the individual combination partners used in ACTs in Bangladesh. However, recent studies conducted in Southeast Asia confirm the need to monitor independently the clinical efficacy and *in vitro* sensitivity of the artemisinin component of ACTs, especially when there are reports of declining efficacy using combination treatment.

In Bangladesh the efficacy of ACTs is generally very good. However, antimalarial drug resistance in Myanmar is high and experience with traditional antimalarials (such as chloroquine and sulfadoxine-pyrimethamine) have shown that drug resistance has a tendency to spread westwards from its origins in Southeast Asia. In 2006/2007 we have conducted studies specifically designed to address reports of failures with ACTs in Southeast Asia, close to the Thai-Cambodian border, using an integrated *in vivo – in vitro* approach. Data from this study suggest that in Southeast Asia there are individual isolates, which may be highly resistant to artemisinins. Although the prevalence of these isolates was low, the overall sensitivity of the parasite isolates was significantly reduced as compared to Bangladesh and even western Thailand, an area with relatively high levels of antimalarial drug resistance, but at the same time an area from which so far there are no reports of increased numbers of failures with artemisinin-based combination therapies (ACTs). In this study individual isolates resulted in increased parasite clearance times, failures after 7 days of artesunate monotherapy, and high inhibitory concentrations for artemisinins. Studies to assess the spread of artemisinin resistance from Southeast to South Asia are therefore urgently needed.

In our study in Cambodia the 28-day cure rate in the artesunate arm (n=60) was 93.3 % (95% CI: 83.8-98.2) vs. 100% (89.7-100%) in the control group (quinine – tetracycline for 7 days following the national treatment guidelines in Cambodia). Mean PCT in patients who failed artesunate monotherapy was almost twice that of cures (101.2 vs. 57.5 hrs; p=0.01) and parasite clearance was significantly prolonged as compared to studies conducted in Bangladesh. At the same time in vitro drug sensitivity tests indicated significantly higher geometric mean IC50s for artemisinins as compared to Bangladesh. Patients who failed therapy had IC50 values up to 10 times higher than the IC50s found in the reference clone W2. Drug levels measured 90 minutes after drug intake on days 0 and 6 were used to define individual dihydroartemisinin (DHA) levels. The mean DHA levels in patients that were cured was 3132.36 (±1830.22) nM on Day 0 and 1645.10 (±823.22) nM on Day 6. In patients that failed therapy the mean drug levels were 2395.21 (±1884.96) and 2074.74 (±1405.83). Out of the 4 patients who failed therapy 2 patients had drug levels higher than the mean on Day 0 and 6, one patient had

drug levels outside of the range of mean ± 1 standard deviations. Although some failures may therefore be linked to inadequate drug levels at least 2 patients (3.33%; 95% CI: 0.41-11.53) with the highest artemisinin IC50s and PCTs of 133 and 95 hrs failed therapy in spite of adequate drug levels suggesting resistance to artemisinins. The aim of the proposed study in southeastern Bangladesh will be to determine the baseline artesunate efficacy and drug sensitivity from an area in which artemisinins have not been used on a large scale.

Artemisinin and its derivatives are the most potent and rapidly acting antimalarial drugs. They reduce the infecting malaria parasite biomass by roughly 10^4 per cycle (White et al. 1999). Once it develops and spreads, resistance to artemisinin derivatives, currently the most essential antimalarial drugs for the treatment of Plasmodium falciparum malaria, could very well be the most devastating event in the history of malaria control in the 21st century (Noedl 2005). More than forty countries have officially adopted artemisinin-based combination therapy (ACT) for the treatment of malaria since 2001. More than twenty alone (including Bangladesh) have adopted the combination of artemether and lumefantrine as their first- or second-line treatment for P. falciparum malaria.

Recent efficacy data from Bangladesh using the combination of artemether and lumefantrine suggest 42-day cure rates of almost 95% (Haque et al. 2007). However, ACTs are starting to fail on both sides of the Cambodian-Thai border. In a recent study the efficacy of the combination of artesunate and mefloquine was reduced to only 79.3% with a 42-day follow-up in Pailin Province (Denis et al. 2006a). Recent reports also suggest increasing numbers of failures with artemisinin combination therapies along the eastern borders of Thailand (Trat Province – data not PCR corrected), potentially due to artemisinin resistance (Vijaykadga et al. 2006). The authors report 21.4% failures in Trat Province with M5AS, a combination of 25 mg/kg mefloquine and 12 mg/kg of oral artesunate (as compared to only 3.4% in Tak province, along the Thai-Myanmar border, an area that is infamous for its high levels of drug resistance).

A possible explanation for higher failure rates particularly in Thailand as compared to many other countries using artemisinin-based combination therapies could be the administration of artesunate as short course over the first 2 days of treatment only. Although the total dose of artesunate (600 mg in adults) used in Thailand is adequate and comparable to most other malaria-endemic nations the drug is administered over only 2 days. Together with the short half-life of artesunate (and its main metabolite dihydroartemisinin) this results in a shorter exposure of the parasites to the drug. However, the effectiveness of artemisinins in reducing the parasite biomass is proportional to the parasite generations that are exposed to the study drug. As the life cycle of P. falciparum is approximately 48 hrs, a 2-day regimen results in only one generation of parasites being exposed to the drug and naturally reduces the efficacy of the drug. However, this cannot explain higher failure rates in eastern as compared to western Thailand as seen in these recent studies.

New ACTs are constantly being developed for the treatment of uncomplicated falciparum malaria. However, even recently introduced ACTs (such as the combination of artemether and lumefantrine) seem to be affected by high failure rates in some regions, either due to absorption problems or drug resistance (Denis et al. 2006b).

In recent years the existence of artemisinin resistance has become a much-discussed issue. Our study in Cambodia was the first to confirm the existence of the first cases of genuine artemisinin resistance using a systematic approach in defining artemisinin resistance. On the other hand suspected clinical artemisinin resistance was reported from Thailand, India, and

Sierra Leone as early as the late 1990s (Luxemburger et al. 1998; Gogtay NJ,et al. 2000; Sahr et al. 2001). Isolated *in vitro* resistance has been reported from numerous countries, particularly in and around Southeast Asia, and artemisinin-resistant strains have been obtained in laboratories by intermittently exposing malaria parasites in culture to rising drug concentrations (Meshnick 2002). However, the significance of many studies reporting artemisinin resistance may be questionable as most reports are limited to either clinical or in vitro data. Generally measuring the clinical impact of antimalarial drug resistance is difficult, and resistance might not be recognized until it is severe. This is partly because routine health information systems may grossly misjudge the magnitude of the problem (Breman 2001). In spite of the fact that few regional drug resistance meetings pass without at least one country reporting either in vitro or in vivo artemisinin resistance, until very recently there has been no convincing evidence for its existence.

Evaluation of suspected cases of artemisinin resistance requires a systematic *in vivo - in vitro* strategy and dose ranging of artemisinins given as monotherapy to assess in how far reduced drug sensitivity can be overcome by higher doses of the artemisinin compound. The only reasonable approach is a careful analysis of clinical treatment response parameters, not just cure rates, combined with *in vitro* drug sensitivity data.

Artesunate for the treatment of malaria

Artesunate has the chemical name (3R,5aS,6R,8aS,9R,10S,12R,12aR)-Decahydro-3,6,9-trimethyl-3,12-epoxy-12H-pyrano[4,3-j]1,2-benzodioxepin-10-ol,hydrogen succinate. Artesunate is a semisynthetic, water soluble, derivative of artemisinin, an antimalarial compound isolated from the Chinese herb Qinghao (Artemisia annua). Artemisinin is a sesquiterpene lactone that bears a peroxide grouping and, unlike most other antimalarials, lacks a nitrogen-containing heterocyclic ring system. Artesunate is the hemisuccinate ester, synthesized by reacting dihydroartemisinin (DHA) and succinic acid anhydride in alkaline medium. This type of reaction invariably yields an ester linkage in alpha configuration. Artesunate is rapidly metabolized to DHA, which is also an active antimalarial, in the body.

As early as 1979, there were several reports on the efficacy of artemisinin against *P. falciparum* and *P. vivax* in over 2000 Chinese patients. It was shown to produce more rapid parasite clearance as compared to other antimalarials and to be highly effective also in chloroquine resistant strains of *P. falciparum*. However, the rate of early recrudescence was high, which was claimed to be due partly to its poor solubility in water and oils and finally led to the development of better soluble derivatives such as the methyl ether derivative (artemether) and the hemisuccinate ester (artesunate) (Karbwang & Na-Bangchang 1993). Chinese physicians tested artemisinin in 6000 patients in the course of the 1970s and the summaries of these studies were finally published in 1982 by the China Cooperative Research Group. At the same time they also reported the first studies with the most important artemisinin derivatives artemether and artesunate in an animal model (Li et al., 1994). It is licensed for use in oral or intravenous preparations throughout Southeast Asia and many African and South American countries, and is first-line therapy for treatment of multi-drug resistant falciparum malaria. The oral drug formulation is on the WHO Essential Drug list. Artesunate is produced by Guilin Pharmaceutical Factory, Guangxi, China and repackaged by Atlantic Laboratories Corp., Ltd., Bangkok, Thailand. The latter is produced under GMP standards. Many million doses have been administered to humans worldwide.

Artesunate: Animal Studies

Artesunate was shown to be highly active against a number of malaria species in animal models. The activity of artesunate and DHA was measured against the erythrocytic forms of *P. falciparum, P. berghei, P. knowlesi, and P. coatneyi* in mice or monkeys. In a preliminary study conducted in immunocompromised mice infected with the T24 strain (chloroquine and quinine resistant) of *P. falciparum*, a complete clearance of parasites from the blood was observed on Day 2 of treatment with DHA (50 mg/kg for 2 days) by the oral route. Microscopic observations at 24 hours showed predominantly pycnotic forms (76%), some altered trophozoites (10%), few trophozoites (1%), and schizonts (3%). At 48 hours, only pycnotic forms were observed. Chloroquine and quinine were not effective in clearing the parasitemia in mice infected with the T24 strain. Treatment with chloroquine induced minimal alterations in morphology (11 - 16% pycnotic forms). However, against a chloroquine sensitive strain (NF54) morphological changes after treatment with chloroquine were similar to that of DHA against the chloroquine-resistant strain. The activity of artesunate was not measured.

In mice infected with *P. berghei* (173N strain) and treated with intravenous artesunate or chloroquine, a 50% and 90% reduction in parasitemia was observed by 18 to 24 hours, and 24 to 30 hours, respectively. Recrudescence was observed on Day 28 in mice treated with artesunate (110 mg/kg) for 5 days by the intravenous route. However, chloroquine (14.9 mg/kg for 5 days) completely cured the mice on Day 28. In another study, mice infected with another strain of *P. berghei* (ANKA strain) showed complete cure on Day 60 after intramuscular treatment with 56 mg/kg artesunate. Complete clearance of the parasitemia was observed within 2 days of treatment. Similar observations were made with DHA. The variation in the activity of artesunate in the different studies may be due to the different strains of *P. berghei* used for infection, the route of drug administration or severity of infection.

In monkeys infected with *P. knowlesi*, the intravenous administration of artesunate (10 mg/kg for 7 days) reduced the parasitemia by 90% at 13 hours. The parasite clearance time (PCT) was 42 hours and all 3 monkeys remained negative for 28 days of observation. However, at the lower dose (3.16 mg/kg), although the PCT was 40 hours, 1/3 monkeys showed recrudescence on Day 15. At a higher dose (31.6 mg/kg), the mean parasite clearance time was 56 hours and no recrudescence was observed. Quinine at 10 mg/kg dose was effective in reducing the parasitemia by 50% in 3.3 hours, however, a 90% reduction of parasitemia was not attained. At a higher dose (31.6 mg/kg), the mean time to parasite clearance was 104 hours and recrudescence was observed 2 to 10 days after parasite clearance.

In another study, normal and splenectomized monkeys infected with *P. coatneyi* were treated with artesunate. The clearance of parasitemia was slower in splenectomized animals compared to normal controls. The reduction in parasitemia at 24 hours in the splenectomized and non-splenectomized animals was 86% and 99%, respectively. Infected erythrocytes showed ultrastructural changes such as enlargement of food vacuoles and ribosomal clumping 4 hours after administration of artesunate. Recently, splenectomized rhesus macaques were tested in a severe malaria model of *P. coatneyi* infection with intravenous artesunate at AFRIMS (RS Miller, unpublished data 2006). Rapid parasite clearance was achieved with optimal dosing noted at 8mg/kg (equivalent to 2.4 mg/kg IV in humans), but recrudescence occurred after many days.

In animal tests, artemisinin compounds are less toxic than quinoline antimalarials. In an oral artesunate study in rats submitted to the FDA for review of rectal artesunate, 3- and 7-day regimens (with a total human equivalent doses of 36 mg/kg) produced no deaths or

neurohistologic lesions (M. Gomez, personal communication 2006). Lethal doses of these compounds in rodents cause multiple system toxicity with bone marrow depression, diarrhea and hemoglobinuria. Sublethal doses produce transient depression of reticulocyte count. 7-day toxicity studies in rhesus macaques found a no effect dose of 8 mg/kg when artesunate was administered for 7 days intravenously. Diarrhea occurred in a dose-dependent fashion at higher doses, usually started after 4-5 days of drug administration. Hemoglobinuria was occasionally seen at 32 mg/kg/day. In lab tests, transient reversible reticulocytopenia was noted at all doses, and elevated alkaline phosphatase was noted at higher doses. Segment 1 reprotoxicity studies in mice reveal no effects on fertility. Segment II studies reveal fetal loss and resorption, particularly during organogenesis in the first trimester.

Animal studies have revealed neurotoxicity in some members of the artemisinin class. Administration of high doses (20 mg/kg/d) of artemether and arteether produces neurotoxicity in rats, dogs and rhesus (gait disturbances, loss of spinal pain responses, restlessness, tremor and incoordination, followed by respiratory depression, convulsions and cardiac arrest) with characteristics brainstem lesions seen on neurohistopathology (Brewer 1994). Results from recent studies demonstrate distinct differences in the ability of artemisinin derivatives to produce neurotoxicity, with fat-soluble derivatives (arteether and artemether) showing much greater propensity to cause these effects. Whereas arteether (25 mg/kg) treated mice showed distinct behavioral changes due to neurotoxicity, behavioral performance was not significantly affected in any rats treated with artesunate (31 mg/kg) (Genovese 2000). The significantly different safety profiles of these drugs may also have to do with the route of administration. Recent studies show that there was no pathologic evidence of neuronal death in mice receiving either oral artemether, or oral or intramuscular artesunate, in doses up to 300 mg/kg/day (Nontprasert et al. 2002). The WHO GLP rat study, IV doses as high as 20 times human equivalent doses, did not produce any clinical neurotoxicity or histologic neuropathology. Likewise, the rhesus study of IV artesunate in Thailand did not reveal any neuropathologic lesions at doses up to 128 mg/kg IV for 7 days (up to 18 times usual human doses). In conclusion, the favorable chemical and pharmacokinetic properties of artesunate suggest that neurotoxicity is not a major concern with this compound.

Artesunate: *In Vitro*

A number of studies conducted in Southeast Asia show that *P. falciparum* parasites remain highly susceptible to artesunate and DHA *in vitro* in most areas, resulting in mean 50% inhibitory concentrations (IC_{50}) for artesunate of 1.24 ng/ml in Vietnam (Wongsrichanalai et al. 1997), 0.35 ng/ml in the Philippines (Bustos et al. 1994), and 0.98 ng/ml in Thailand respectively (Noedl et al. 2004). Although the IC50s found in different regions of the world showed considerable variations in their levels of sensitivity, until very recently there was little evidence towards a developing artemisinin-resistance (Wongsrichanalai et al., 1999; Ringwald et al., 1999). Recent *in vitro* as well as *in vivo* data from Thailand and other Southeast Asian countries however suggest increasing numbers of failures and higher IC50s.

Artesunate: Efficacy and Safety Profile

Artesunate (AS) has been licensed for the treatment of malaria in many Southeast Asian countries since the 1990s. The drug is well absorbed, and rapidly hydrolyzed in the liver to the more active metabolite, dihydroartemisinin. Its half-life is less than 1 hour, so that all biological activity is gone after an oral dose within 12 hours (Teja-Isavadharm et al., 2001). While this drug is currently still considered to be the most potent blood schizonticide available, this rapid

clearance requires repeated dosing of artesunate in order to clear all metabolically susceptible parasites. Generally artemisinin derivatives (artesunate and artemether) are extremely well tolerated when used at therapeutic dosage (Price et al. 1999).

Data from 23 trials with 1891 patients (Hien and White 1993) comparing artemisinin derivatives with other antimalarials showed shortening of fever clearance time compared to intravenous quinine and parasite clearance time by 17 and 32% respectively. Artesunate appeared to have more rapid action than the other derivatives. No serious toxicity was observed in these trials. 600 mg at 0 h and 4 h followed by 400 mg at 24, 32, 48 and 56 h was compared with oral quinine 1500 mg daily in 3 divided doses over 14 consecutive days, in patients with acute malaria. Shorter parasite and fever clearance times occurred in the artemisinin group but 50% of patients recrudesced compared with 23% in the quinine group. Monotherapy with artesunate is ineffective when given for 3 days or less. Li (1994) summarized the early Chinese data, reporting a recrudescence rate of 51% when artesunate monotherapy was administered for 3 days (total dose 280 mg-400mg) to 65 adults with uncomplicated falciparum malaria. Shorter regimens (total dose AS 600mg) given over 1-2 days did not cure any volunteers in Thailand (Bunnag et al. 1991). A subsequent randomized controlled trial by Li (1997) showed 39% recrudescence rate at day 28 with 400mg given over 3 days.

Extending the duration of treatment with artesunate has significantly improved the efficacy. Li (1994) reported a recrudescence rate at day 28 of 5 % when artesunate (Guilin) was given for 5 days (total dose of 440mg-600mg) to 144 patients with uncomplicated falciparum malaria in China, and 7% recrudescence in his subsequent study (600 mg over 5 days). Studies in Thailand using 600mg over 5 days confirmed these findings with 84% cure rates at day 28 in 167 volunteers (Looareesuwan et al. 1994). A study conducted by the same group suggests cure rates of 98 to 100% for 7 and 5 days respectively of artesunate treatment when using overall doses of 1200 to 1600 mg of artesunate (Looareesuwan et al. 1997). The treatment was well tolerated and found to be safe. These studies led to more extensive combination testing with other antimalarials, which now have become standard practice.

Artesunate Safety Data

Price and colleagues (1999) reviewed the cumulative experience of the Shoklo Malaria Research Unit, Thailand, with over 3500 cases of artemisinin derivatives, mostly oral artesunate. Vomiting occurred in 2.2% of those receiving artemisinins alone the first day, with a significant risk associated with prior vomiting or nausea before enrollment (OR=2.8 and 2.1, respectively). The incidence of vomiting fell with subsequent days.

No seizures were reported in artesunate monotherapy groups, but 15 seizures following treatment with mefloquine and artesunate/artemether (incidence 177/100,000). No urticaria, hemoglobinuria, or neuropsychiatric reactions occurred in AS monotherapy treated persons (n=836). Lab tests showed no significant changes in WBC or neutrophil counts, although platelets did drop in 2 of 154 volunteers (AS + MEF) during treatment, with resolution by day 14. No renal dysfunction occurred in any patient, but 4.5% experienced some increase in liver transaminases with no clinical hepatic dysfunction (all co-treated with mefloquine). No significant changes were seen on EKG during therapy, including tests 1 hour after artesunate administration. Case reports with artesunate in persons with malaria have rarely recorded bradycardia, 1° AV block or QT prolongation, but all are flawed by concurrent malaria which can cause these findings due to hypocalcemia.

A neurologic exam (consisting of fine-finger dexterity, heel-toe walk, hearing assessed by tuning fork, eye exam for nystagmus and Rombergs test) was performed on admission, day 2, day 7 and day 28 in 1,664 volunteers receiving artesunate (Price et al. 1999). Dizziness was the most common complaint, with exam evidence of disturbed balance in 5 of 690 treated with artemisinins alone. All signs and symptoms resolved by day 7. No one developed deafness or permanent neurologic injury. A multicentre trial conducted in Africa in 941 children (age 10 years or older) with randomly assigned amodiaquine plus artesunate or amodiaquine and placebo suggests that artesunate combination therapy is also safe in children (Adjuik et al. 2002). A combination of artesunate with sulfadoxine-pyrimethamine was found to be safe, well tolerated, and efficacious in children with uncomplicated falciparum malaria in The Gambia (Doherty et al. 1999; von Seidlein et al.2000). A case-control study from Thailand failed to detect any evidence of significant neurotoxicity in 79 patients treated previously with oral artemether or artesunate for acute malaria (Van Vugt 2000), and Phase IV monitoring by the WHO has failed to reveal neurotoxicity in any humans treated with artesunate (M Gomes, personal communication 2006).

A prospective trial conducted in Thailand in 461 pregnant women suggested that artesunate was well tolerated and that birth outcomes did not differ significantly to community rates for abortion, stillbirth, congenital abnormality, and mean gestation at delivery (McGready et al. 2001). An earlier study conducted by the same group found no congenital abnormality in any of the newborn children whose mothers were treated with artesunate for falciparum malaria during pregnancy, and that all children followed for more than one year developed normally (McGready et al.1998). Nonetheless, based on the animal data, the drug is contraindicated in the first trimester of pregnancy.

Study area

With an area of 501.99 sq km Bandarban Sadar Upazila (Bandarban district) is bounded by Rajasthali upazila to the north, Lama upazila to the south, Rowangchhari and Ruma upazilas to the east, Rangunia, Chandanaish, Satkania and Lohagara upazilas to the west. Bandarban has a humid tropical climate and is mostly made up of hill ranges that provide an excellent breeding ground for malaria vectors.

The area of Bandarban municipality is 51.80 sq km. It has a population of 31806; male 60.22%, female 39.78%. It has 10 wards and 62 mahallas. There are 2 dakbungalows. Literacy rate among town people is 56.8%. Bandarban thana was established in 1923 and was turned into an upazila in 1983. It consists of 8 union parishads, 63 mouzas and 199 villages.

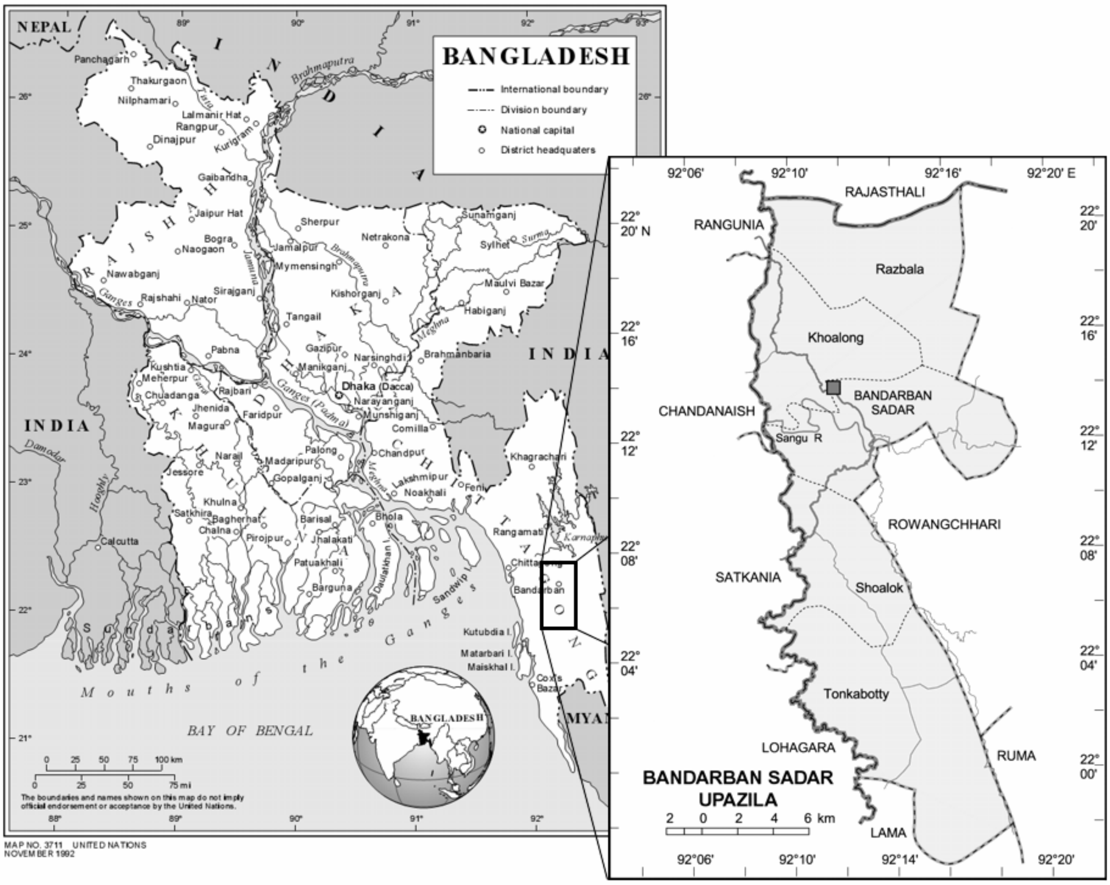

Malaria is a major health burden in this remote, mountainous southeastern region of Bangladesh. The official number of laboratory confirmed malaria cases reported from Bangladesh is only 56,879 with more than 500 laboratory confirmed deaths occuring due to malaria annually. However, the number of unreported and clinically diagnosed malaria cases is estimated to range somewhere between 400,000 and 1 million with *Plasmodium falciparum* as the dominant species (70% of all cases) (WHO, World Malaria Report 2005).

WHO concludes that the malaria situation in Bangladesh is worsening, particularly in the hilly and forested areas in the Hill Tract Districts of the Southeast, where this study will be conducted, and also along the border areas in 13 high endemic districts with reported chloroquine and sulfadoxine/pyrimethamine resistance. Recently the Ministry of Public Health has revised the malaria treatment regimen with the introduction of Artemisinin-based combination therapy in the reported drug resistant areas. However, surveillance is weak in these areas as a result of difficult terrain and inaccessibility. There is a shortage of staff and doctors in health facilities as well as significant lack of research data about malaria. Increasing drug resistance is significantly aggravating the malaria situation and treatment alternatives to currently used antimalarials are urgently needed.

Preliminary data during the period Sept 2002 to Dec 2005 from BRAC's malaria pilot program in this region shows:

Uncomplicated malaria treated: 136,926

Treatment failure malaria treated:	6,319
Blood tested:	21,768
Malaria positive:	9,678 (44%)

Standards of health care for acute uncomplicated P. falciparum malaria in Bangladesh

Standard of care (first line therapy) for uncomplicated P. falciparum malaria is the combination of artemether and lumefantrine. However, due to financial constraints chloroquine remains a commonly used therapy for uncomplicated falciparum malaria in Bangladesh.

Table 1: A six-dose regimen of the combination of artemether with lumefantrine is used as the first line therapy for the treatment of uncomplicated *P. falciparum* malaria in Bangladesh.

Weight (w)	D1 (Tablets)	D2 (Tablets)	D3 (Tablets)
5-15 kg	2 x 1	2 x 1	2 x 1
15-25 kg	2 x 2	2 x 2	2 x 2
25-35 kg	2 x 3	2 x 3	2 x 3
≥35 kg	2 x 4	2 x 4	2 x 4

Quinine given 3 times daily over 7 days is the official second line therapy for uncomplicated falciparum malaria in Bangladesh.

2.2 Rationale

Artemisinin-based combination therapies (ACTs) have recently been introduced as the official first line therapy for uncomplicated falciparum malaria in Bangladesh. However, recent data indicate reduced overall sensitivity of *P. falciparum* to artemisinin derivatives both *in vivo* as well as *in vitro* in Southeast Asia. In our recent studies (Artemisinin Resistance in Cambodia I) in this region individual isolates were detected that are highly resistant to artemisinins. Reports from the Ministries of Public Health on both sides of the Thai-Cambodian border indicate increasing numbers of treatment failures with artemisinin-based combination therapies, currently the last line of defense against the spread of multidrug resistant malaria (Denis et al. 2006a; Vijaykadga et al. 2006). The efficacy of ACTs in Bangladesh is still high (unpublished data 2007). However, no data are available on the efficacy of the individual combination partners.

The study will be conducted in Bandarban District, in the Chittagong Hill Tracts, the area and with the population most affected by malaria in Bangladesh. Adequate efficacy with combination regimens do not necessarily indicate that both combination partners are still

adequately active. An integrated *in vivo-in vitro* approach will be used to distinguish drug resistance from drug failures.

The proposed study will assess whether failures with high-dose artemisinin treatment can be expected even in areas where artemisinins have never been used on any significant scale. This study will help to understand whether artemisinin-resistant parasites are inherently part of Asian parasite population or whether artemisinin resistance is induced by extended use of artemisinins (drug pressure). The 2 artemisinin monotherapy arms will provide data on dose-dependent efficacy. The study will also provide important baseline efficacy data for artemisinins in Bangladesh that will allow for a longitudinal surveillance of artemisinin sensitivity in the region. In terms of antimalarial drug resistance surveillance Bangladesh is of particular importance as it serves as a gateway for the Indian subcontinent.

Artesunate monotherapy is not recommended for the treatment of *P. falciparum* malaria. Currently artemisinins are being used in combination therapy for the treatment of uncomplicated falciparum malaria (artemether – lumefantrine). Artesunate monotherapy will only be used in this study to identify potential artemisinin resistance in Bangladesh. Even a very high efficacy of artesunate monotherapy should not lead to policy implementation in Bangladesh.

2.3 Potential Risks and Benefits

The risks for the patients are expected to be particularly low as all treatments used in this trial are expected to be highly efficacious and safe.

2.3.1 Potential Risks

The subject may experience a brief moment of physical discomfort or pain during the finger prick procedure and the venipuncture and there is a possibility of bruising and/or infection at the site of the finger-prick or venipuncture.

Subjects may experience side effects from the study drugs. Artesunate is generally very well tolerated but may occasionally cause mild abdominal symptoms and dizziness and changes in the laboratory and EKG parameters. Quinine is considered to be one of the safest drugs available for the treatment of falciparum malaria but frequently causes characteristic side effects due to cinchonism (in some studies up to 100% of patients report side effects due to cinchonism).

There is a risk that malaria may recur. All subjects will therefore be closely monitored and quickly treated should any signs or symptoms of malaria be detected.

2.3.2 Potential Benefits

As subjects will be hospitalized for at least the first 7 days and thereafter closely monitored for any signs and symptoms of malaria they will have the benefit of close medical supervision. All doses of the study drugs will be administered under direct supervision by medical/nursing staff trained in drug administration, and any change in

the course of their infection or any adverse experiences will be recognized and treated more rapidly than would normally occur if they were treated as outpatients in a government facility.

Subjects will be immediately treated for any reappearance of parasitemia that occurs. Subjects who fail initial therapy, based on parasitological parameters, will be treated with a standard regimen following the national treatment guideline that is known to be effective. In addition, while admitted to the hospital the subjects may be examined and treated for other concurrent illnesses. Subjects will also receive medical attention and appropriate standard medical care or referral should they become ill during the study.

An early detection of artemisinin resistance and the knowledge whether failures can be overcome by increasing the dosage of artemisinins will directly benefit the malaria control program and the population of Bangladesh and will allow for adequate countermeasures to be taken in case artemisinin resistance should be detected. The national malaria program of Bangladesh will benefit from human resource capacity building, access to modern laboratory methodology (particularly novel techniques for *in vitro* drug sensitivity testing of antimalarial drugs) and training.

3 OBJECTIVES

3.1 PRIMARY OBJECTIVE:

- Provide baseline efficacy data for artemisinins in Bangladesh that will allow for a longitudinal surveillance of artemisinin sensitivity.

3.2 SECONDARY OBJECTIVES:

- To assess whether artemisinin-resistant parasites are inherently part of Asian parasite population or whether artemisinin resistance is induced by extended use of artemisinins (drug pressure).
- Validate treatment response parameters (PCT, FCT) for their role in predicting failures.
- To determine the genetic background of artemisinin sensitivity in *Plasmodium falciparum*.

4 STUDY DESIGN

This is an open label, randomized study. The study design is largely based on the WHO recommendations for the 'Assessment and Monitoring of Antimalarial Drug Efficacy for the Treatment of Uncomplicated Falciparum Malaria' (WHO, 2003). Patients with acute uncomplicated falciparum malaria will be randomly assigned to one of 3 arms: All patients in arm 1 and 2 will receive artesunate monotherapy for 7 days (Table 5). Patients in the control group will receive quinine and doxycycline for 7 days. The ratio of enrollment into the 3 groups will be 2:2:1. Arm 3 serves as a control. The sample size in arm 3 is lower as historical controls from other sites in Asia (particularly Cambodia, where drug resistance is generally higher and were 100% cure rates were determined for the combination of quinine with tetracyclines given over 7 days – Noedl, data to be published) can be used.

Table 5: Antimalarial drug dosing for treatment groups 1-3.

Treatment Group	Sample Size	Artesunate		Quinine		Doxycycline	
		Dosing mg/kg/day	Total Dose/kg	Dosing mg/kg/day	Total Dose/kg	Dosing mg/kg/day	Total Dose/kg
1 (Artesunate)	40	2	14	N/A	N/A	N/A	N/A
2 (Artesunate)	40	4	28	N/A	N/A	N/A	N/A
3 (Controls)	20	N/A	N/A	30	210	4	28

Any drug dose vomited within 60 minutes after drug administration will be repeated. Patients will be admitted to the hospital for the duration of study drug administration or until all signs and symptoms of malaria have disappeared, whichever is longer. Patients will be asked to return for follow-up visits on days 7, 14, 21, 28, 35, and 42. Blood will be drawn on the day of admission (before initiating therapy) for *in vitro* drug sensitivity testing and for PCR (markers of drug resistance and to distinguish recrudescence from reinfection by genotyping). Malaria smears will be prepared twice daily (up to 4 times if clinically warranted) until PCT and on Days 7, 14, 21, 28, 35, and 42 or whenever symptoms consistent with malaria appear. Plasma samples for determining drug levels will be obtained on Days 0 and 6. Over the entire study, up to approximately 32 ml of blood may be drawn by venipuncture from patients who do not develop recrudescences. An additional 8 ml of blood will be drawn in case of recrudescence. The study duration for each individual patient will be 42 days.

All patients in group 1, 2, and 3 will receive antimalarial therapy for 7 days for the treatment of their *P. falciparum* infection. Patients who develop early treatment failures, who fail to completely clear parasites by Day 7 or who have a recurrence of parasitemia any time until Day 42 will be treated with 1st or 2nd line therapy following the national treatment guidelines of Bangladesh.

Study drug:

Artesunate

Chemical name:	Artesunate has the chemical name (3R,5aS,6R,8aS,9R,10S,12R,12aR)-Decahydro-3,6,9-trimethyl-3,12-epoxy-12H-pyrano[4,3-j]1,2-benzodioxepin-10-ol,hydrogen succinate
Generic name:	Artesunate
Trade name:	Artesunate, Artesunet
Dosage form:	Tablet
Strength:	50 mg
Source:	Supplied by the World Health Organization or local purchase Bangladesh

Quinine and doxycycline will be purchased locally.

All study drugs will be stored at room temperature in a locked cabinet under controlled conditions to ensure drug quality and stability.

Treatment failures (reemergence of parasitemia):

Patients who fail initial therapy in the artesunate arms (arm 1 or 2) will be treated with 7 days of quinine (2nd line therapy according to the national treatment guideline)

Patients who fail initial therapy in the control arm (arm 3) will be treated with 3 days of Coartem (1st line therapy according to the national treatment guideline)

5 STUDY POPULATION

5.1 Selection of the Study Population

Subjects will be male and non-pregnant female patients (age 8 - 65 years), recruited by passive or routine active case detection at Bandarban Sadar Hospital and Bandarban District, Bangladesh, who present with acute uncomplicated falciparum malaria. Children and adolescents will be included as this is the population most affected by falciparum malaria in Bangladesh. Uncomplicated falciparum malaria is defined as falciparum malaria in the absence of any signs or symptoms consistent with severe malaria as defined by WHO (WHO 2000). No additional recruitment material will be used in patient recruitment. Subjects diagnosed with uncomplicated Pf malaria upon evaluation will be verbally notified about the ongoing trial, and asked to participate if interested. Study personnel will screen all persons reporting that they want to take part in the study, give study information and informed consent to the subject and screen for inclusion/exclusion criteria. The patients will be given the choice of either receiving standard of care treatment for their malaria or participating in the study. If potential volunteers cannot read the consent form, study personnel will verbally review the study information/consent form with them and answer all questions.

5.2 Study Site

The study will be performed in Bandarban District, Southeastern Bangladesh. Inpatient care will be provided at the Bandarban Sadar Hospital under direct supervision of the investigators. The local personnel is trained and has experience in recruiting and providing inpatient care to study subjects from previous clinical trials.

5.3 Inclusion Criteria

Male and female subjects with a diagnosis of acute falciparum malaria meeting all criteria listed below may be included in the study:

1. Acute symptomatic falciparum malaria infection as determined by malaria smear with a parasite density of 1,000 to 100,000 asexual parasites/µL as determined on the screening smear with fever (defined as ≥37.5ºC), or reported history of fever within the last 48 hours.

2. Age: 8-65 years old

3. All females between the age of 12 and 50 are required to have a negative human chorionic gonadotropin (hCG) pregnancy test (urine). All females of childbearing

potential (not surgically sterile, or less than two years menopausal) are required to use an acceptable method of contraception throughout the study

4. Written informed consent obtained
5. Willing to stay under close medical supervision for the study duration of 42 days
6. Otherwise healthy outpatients

5.4 Exclusion Criteria

Subjects presenting with any of the following will not be included in the study:

1. Pregnant women, nursing mothers, or women of childbearing potential who do not use an acceptable method of contraception
2. Mixed malaria infection on admission by malaria smear
3. A previous history of intolerance or hypersensitivity to the study drugs or to drugs with similar chemical structures
4. Malaria drug therapy administered in the past 30 days by history
5. History of significant cardiovascular, liver or renal functional abnormality or any other clinically significant illness, which in the opinion of the investigator would place them at increased risk.
6. Symptoms of severe vomiting (no food or inability to take food during the previous 8 hours).
7. Signs or symptoms of severe malaria (as defined by WHO 2000)
8. Unable and/or unlikely to comprehend and/or follow the protocol

5.5 Randomization Procedures

Subjects will be assigned unique identification codes in order of inclusion and randomly assigned to either one of the artesunate groups. The ratio of allocation to the three groups is 2:2:1. Randomization will not be stratified and will be done using random number tables in blocks of 10. A randomization list will be created prior to beginning the trial. Sealed individual envelopes that will be opened only on enrollment of each subject will be used. The study personnel enrolling the subject will therefore not know which group the patient will be assigned to until opening the envelope. The randomization process will continue until the minimum sample size of evaluable subjects has been reached in all arms.

6 STUDY PROCEDURES/EVALUATIONS

6.1 Study Procedures

The detailed design is outlined below and in the Appendices. Patients will be admitted to the Bandarban Sadar Hospital for the duration of study drug administration or until all signs and symptoms of malaria have disappeared, whichever comes later. Patients will be asked to return for follow-up visits on days 7, 14, 21, 28, 35, and 42. Follow-up as an outpatient is until Day 42.

As compared to the conventional treatment of uncomplicated falciparum malaria, which involves treatment in an outpatient setting or hospital admission for only 1-3 days the patient will be admitted for a minimum of 7 days. Conventional patient care also involves only relatively few blood draws for routine diagnostics. For study participants the number of blood draws will be considerably increased (see section 6.2).

At the screening/baseline visit, the study will be fully explained to the subjects and written informed consent obtained. Subjects will be assessed as to whether they meet the inclusion/exclusion criteria. Demographic details (age, sex, weight, height) and medical history and concomitant medication details will be recorded. Vital signs (pulse, blood pressure, respiratory rate, and temperature) will be taken. A physical exam will be performed and clinical signs and symptoms will be evaluated. Blood smears to confirm *P. falciparum* malaria will be performed. Blood samples will be collected following the schedule outlined below.

Patient response to therapy will be monitored by assessing clinical parameters at baseline (Day 0) and daily at least until peripheral blood smears are negative for parasites (PCT). During hospitalization patients will have smears performed twice daily until the smears are negative on 2 successive blood smears. Thereafter blood smears will be performed on Days 7, 14, 21, 28, 35, and 42. A final physical examination and blood testing may be requested from subjects who decide to withdraw from the study.

6.2 Laboratory Evaluations

Total Amount of venous blood to be drawn: 40 ml (max)

	Chemistry/ Hematology	In vitro drug sensitivity	Molecular analysis	Pharmaco-kinetics
Day 0	4 ml	4 ml	4 ml	4 x 2ml
Day 3	4 ml			
Day 6				4 x 2ml
Recrudescence		4 ml	4 ml	

6.2.1 Laboratory Evaluations/Assays

- Hematology (hemoglobin, RBCs, WBCs, platelets) on days 0, 3 (or whenever clinically warranted). Additional parameters may be assessed if clinically warranted.

- Blood chemistry on days 0 and 3 (or whenever clinically warranted): blood glucose, creatinine, total bilirubin. Hematology and blood chemistry will be performed at the research laboratory at the Bandarban Sadar Hospital. Additional parameters may be assessed if clinically warranted.

- Urine pregnancy test: Urine beta HCG test. All female subjects older than 12 years will undergo a pregnancy test at baseline. Pregnant women will not be eligible for entry into the study.

- Peripheral blood smears from finger prick blood on day 0 and then daily (up to every 6 hours) until PCT:
    - To determine species of parasite
    - To quantify parasitemia

6.2.2 Special Assays or Procedures

- Blood sample for parasite DNA fingerprinting, RNA, and *in vitro* drug sensitivity will be collected.

    - Samples for in vitro drug sensitivity testing (4 ml) will be collected on Days 0 and in case of reemergence of parasitemia. HRP2 *in vitro* drug sensitivity assays will follow the routine established previously (Noedl 2004, 2005)

    - Samples for DNA fingerprinting (4 ml) will be collected on Day 0 and in case of recrudescence to distinguish reinfections from recrudescences and to define markers of drug resistance. Fingerprick blood will be collected on filter paper for parasite RNA.

- Plasma/serum samples for determining drug levels (2 ml of whole blood per blood draw) will be collected from all patients in arms 1 and 2 on Days 0 and 6 just before and 60, 120, and 180 (±15 minutes) after drug intake.

6.2.3 Specimen Collection, Preparation, Handling and Shipping

6.2.3.1 Specimen Preparation, Handling, and Storage

Stained thick and thin blood smears will be examined by a microscopist who is blinded to the treatment status of the study subject. Microscopists will be blinded to the clinical picture of the subject and to each others results. All slides that are positive for recrudescence will be reexamined by an expert microscopist. In case

of a difference in results (positive/negative; species diagnosis) between the two microscopists, the blood smear will be re-examined by a third microscopist and the third reading will be accepted as the final result.

Parasite densities will be calculated based on a count of parasites per 200 WBCs (thick film) or per 2000 RBCs (thin film). A total of 200 oil immersion fields will be examined before a blood smear is considered negative. A random subset of the stained microscopy slides will be re-examined by independent microscopists (e.g. USAMC-AFRIMS, Bangkok) for quality assurance purposes.

Blood samples for DNA fingerprinting will be collected in EDTA tubes and stored frozen.

Blood samples for *in vitro drug* sensitivity testing will be stored refrigerated and the HRP2 drug sensitivity assays will be performed directly at the study site. The remaining blood will be stored in liquid nitrogen for future drug sensitivity analysis.

The whole blood drawn for the measurement of drug levels (PK) will be centrifuged immediately and the plasma will be stored frozen until testing. The frozen plasma will be stored at the study site until being transported to the respective laboratory for analysis.

6.2.3.2 Specimen Shipment

Blood specimens collected on site will be stored under supervision and responsibility of both PIs at the field site in Bandarban, the ICDDR,B or the Medical University of Vienna until further testing. Samples will be jointly owned by the institutions involved. Samples may only be used for additional tests with the approval of both PIs. The amount of blood collected will only be used for the tests outlined in this protocol. No blood will be stored for human testing. Parasite samples may be stored for up to 20 years before being destroyed. Samples will not be used for HIV or human genetic testing. Blood samples will only be used for purposes outlined in the protocol (malaria drug resistance research). If tests other than those specified in the protocol should be performed, all IRBs will be notified.

The tests will be performed on site (HRP2 drug sensitivity assay, microscopy, hematology, biochemistry, and urine pregnancy test) or at the ICDDR,B, the laboratories of the Medical University of Vienna or at external laboratories to be named. Specimens will regularly be shipped to the laboratories for analysis.

As this study is part of a consortium effort coordinated by the World Health Organization (WHO) in Geneva samples will be shipped to external laboratories for testing. Markers of drug sensitivity and parasite genetic testing will be established by the University of Maryland (Dr. C. Plowe). In vitro activity of cryopreserved parasite culture samples will be established at the University of South Florida (Dr. D. Kyle). All external laboratory work will be coordinated and supervised by the WHO.

7 STUDY SCHEDULE

Appendix A *Schedule of Procedures/Evaluations* contains the detailed schedule of observations and assessments to take place during the study.

7.1 Screening

At the screening/baseline visit, the study will be fully explained to the subjects and written informed consent obtained from the subjects.

Subjects will be assessed as to whether they meet the inclusion/exclusion criteria. Patient who meet all criteria will be enrolled. Demographic details (age, sex, weight, height) and medical history and concomitant medication details will be recorded. Vital signs (pulse, blood pressure, respiratory rate, and temperature) will be taken. A physical exam will be performed and clinical signs and symptoms will be evaluated. Blood smears to confirm *P. falciparum* malaria will be performed. A blood sample will be taken for hematology and blood glucose analysis and a urine sample provided.

All female subjects between the age of 12 and 50 years will undergo a urine pregnancy test at baseline. Pregnant women will not be eligible for entry into the study. They should either abstain from sexual relations or practice an acceptable method of contraception (including "modern contraception" i.e. IUDs, hormonal contraception and barrier methods like condoms). Counseling will be provided to women of childbearing potential and if they wish to participate will be offered an acceptable method of contraception free of charge for the duration of their participation in the study. Should pregnancy be suspected during the study the study subjects are advised to immediately notify the investigator. Should a woman become pregnant during the first 2 weeks of her participation in this study she will be provided with pregnancy care and will be followed up until delivery to record pregnancy outcomes.

Blood samples will be drawn for parasite DNA fingerprinting and *in vitro* drug sensitivity analysis. Patient response to therapy will be monitored by assessing clinical parameters at baseline (Day 0) and daily at least until peripheral blood smears are negative for parasites. Plasma samples for determining drug levels will be collected from all patients in arms 1 and 2 at baseline and on Day 6.

7.2 Follow-up and Final Visits

Patients will be admitted to the hospital for the duration of study drug administration (7 days) or until all signs and symptoms of malaria have disappeared, whichever is longer. Thereafter patients will be asked to return for follow-up visits on days 7, 14, 21, 28, 35, and 42. Costs incurred by participants due to their participation in the study will be reimbursed.

During hospitalization patients will have malaria smears performed twice daily until the smears are negative on 2 successive blood smears. Thereafter blood smears will be performed on Days 7, 14, 21, 28, 35, and 42 or whenever signs and symptoms consistent with malaria reappear.

On Day 3 hematology and blood chemistry will be repeated (or whenever clinically warranted).

The time windows for the follow-up visits on Days 14, 21, and 35 are -2 to +6 days. Those on Days 7, 28, and 42 are 0 to +6 days. Patients who do not attend the study site for follow-up will be contacted at their homes by study staff.

To maximize the scientific integrity of the study and patient safety, every effort will be made by study staff to contact patients missing follow up appointments. This may include telephone contact with subjects or visits to patient homes. Sample size calculations take into account a projected loss rate based on previous experience. Data and samples accrued from subjects subsequently lost to follow up will be stored and handled in the same fashion as the larger database including data from participants completing the study.

Patients who are found to have blood smears positive for *P. falciparum* malaria during follow-up will be treated according to national treatment guidelines. Patients who develop P. vivax parasitemia during follow-up will be censored in the analysis.

7.3 Criteria for Discontinuation or Withdrawal of a Subject

Any subject may be discontinued from the study at any time at the discretion of the investigator if he/she feels it is in the best interest of the subject or if in the judgment of the investigator continuing in the study would be harmful and/or inappropriate for the subject (e.g. patients not tolerating the study drug, development of SAEs that require referral to another hospital) or if a patient cannot be followed thereby not permitting adequate safety assessment. Women who become pregnant during the trial will be taken off the study medicine and will be treated following the national treatment guidelines. If a woman becomes pregnant after the first 7 days she will be withdrawn from the study and will be followed for safety.

8 ASSESSMENT OF OUTCOME MEASURES

8.1 Specification of the Appropriate Outcome Measures

Outcome measures for the clinical study is based on the criteria set forth by WHO in the "Assessment and Monitoring of Antimalarial Drug Efficacy for the Treatment of Uncomplicated Falciparum Malaria" for low to moderate transmission areas (WHO, 2003).

8.1.1 Primary Outcome Measures (Clinical Outcomes)

Clinical outcome will be assessed separately for Day 28 (uncorrected) and 42 (PCR-corrected). Classification of clinical outcomes:

- **Adequate Clinical and Parasitological Response (ACPR)**
  • Absence of parasitemia on Day 28 and 42 irrespective of axillary temperature without previously meeting any of the criteria of Early Treatment Failure or Late Clinical Failure or Late Parasitological Failure.

- **Early Treatment Failure (ETF)**
  • Development of danger signs (e.g. impaired conciousness, convulsions, respiratory distress) or severe malaria on Day 1, Day 2 or Day 3, in the presence of parasitemia
  • Parasitemia on Day 2 higher than Day 0 count irrespective of axillary temperature
  • Parasitemia on Day 3 with axillary temperature ≥ 37.5 °C
  • Parasitemia on Day 3 ≥ 25 % of count on Day 0.

- **Late Treatment Failure (LTF)**
  - Late Clinical Failure (LCF)
    • Development of danger signs or severe malaria after Day 3 in the presence of parasitemia, without previously meeting any of the criteria of Early Treatment Failure
    • Presence of parasitemia and axillary temperature ≥ 37.5 °C (or history of fever) on any day from Day 4 to Day 28/42 without previously meeting any of the criteria of Early Treatment Failure
  - Late Parasitological Failure (LPF)
    • Presence of parasitemia on any day from Day 7 to Day 42 and axillary temperature < 37.5 °C, without previously meeting any of the criteria of Early Treatment Failure or Late Clinical Failure

8.1.2 Primary Outcome Measures (Laboratory Outcomes)

Classification of laboratory outcomes.

- **Inhibitory Concentrations (ICs)**

- Inhibitory concentrations will be calculated by nonlinear regression analysis. The outcome is a continuous variable.

8.1.3 Secondary Outcome Measures

The secondary clinical endpoints are time until full clearance of parasites (parasite clearance time, PCT), gametocytes (gametocyte clearance time, GCT), and to disappearance of fever (fever clearance time, FCT: the time from start of treatment until the axillary temperature drops to below 37.5 °C and remains below this temperature during the next 48 hours). The event time for clearance times is defined as the time until the first in a series of negative tests occurs. PCT, GCT, and FCT are continuous variables.

9 SAFETY ASSESSMENT AND REPORTING

Adverse event assessment will be done throughout the study duration of 42 days. A medical monitor will be assigned and an established internal monitoring and quality control process will be used for regularly monitoring the safety of the patients and the quality of the collected data (at least every 3 months).

9.1 Definition of Adverse Event (AE)

An AE is defined as any untoward medical occurrence in a subject undergoing a study related procedure and believed reasonably to be caused by that study related procedure.

9.2 Definition of Serious Adverse Event (SAE)

An SAE is any untoward medical occurrence regardless of cause or relationship to study drug that:

- Results in death.
- Is life-threatening. Any adverse experience that places the subject, in the view of the investigator, at immediate risk of death from the reaction as it occurred (i.e., it does not include a reaction that, had it occurred in a more serious form, might have caused death).
- Requires in-patient hospitalization (excluding the hospitalization required by the study) or prolongation of existing hospitalization.
- Results in persistent or significant disability or incapacity.
- Is a congenital anomaly/birth defect.
- An event that required intervention to prevent permanent impairment or damage.
- Important medical events that do not result in death, are not life-threatening, or do not require hospitalization may be considered serious adverse events when, based upon appropriate medical judgment, they might jeopardize the subject and might require medical or surgical intervention to prevent one of the outcomes listed above.

9.3 Safety Reporting Procedures

All observed or volunteered adverse events regardless of treatment group or suspected causal relationship to study drug will be recorded on the adverse event pages of the case report form. Events involving adverse drug reactions, illnesses with onset during the study, or exacerbations of pre-existing illnesses other than malaria should be recorded. Exacerbation of pre-existing illness, including the disease under study, is defined as a manifestation (sign or symptom) of the illness that indicates a significant increase in the severity of the illness as compared to the severity noted at the start of the trial. It may include worsening or increase in severity of signs or symptoms of the illness, increase in frequency of signs and symptoms of an intermittent illness,

or the appearance of a new manifestation/complication. Exacerbation of a pre-existing illness should be considered when a patient/subject requires new or additional concomitant drug or non-drug therapy for the treatment of that illness during the trial. Lack of or insufficient clinical response, benefit, efficacy, therapeutic effect, or pharmacologic action should not be recorded as an adverse event. The investigator must make the distinction between exacerbation of pre-existing illness and lack of therapeutic efficacy. In addition, clinically significant changes in physical examination findings and abnormal objective test findings (e.g., laboratory) should also be recorded as adverse events. The criteria for determining whether an abnormal objective test finding should be reported as an adverse event are as follows:

- test result is associated with accompanying symptoms, and/or
- test result requires additional diagnostic testing or medical/surgical intervention, and/or
- test result leads to a change in study dosing or discontinuation from the study, significant additional concomitant drug treatment or other therapy, and/or
- test result leads to any of the outcomes included in the definition of a serious adverse event, and/or
- test result is considered to be an adverse event by the investigator

Merely repeating an abnormal test, in the absence of any of the above conditions, does not meet conditions for reporting as an adverse event.

Any abnormal test result that is determined to be an error does not require reporting as an adverse event, even if it did meet one of the above conditions except for condition #4.

9.3.1 Serious Adverse Event Detection and Reporting

All serious adverse events will be

- recorded on the appropriate serious adverse event case report form
- followed through resolution by a study physician
- and reviewed by a study physician.

Unanticipated problems involving risk to subjects or others, serious adverse events related to participation in the study and all subject deaths should be promptly reported to the IRB of record:

Ethical Review Committee
International Centre for Diarrhoeal Disease Research, Bangladesh
Mohakhali, Dhaka-1212, Bangladesh
Tel.: +(880-2) 9886498
Fax: +(880-2) 882 3116

The study clinician will complete a Serious Adverse Event Form within the following timelines:

- All deaths, whether associated or not associated, will be recorded on the Serious Event Form and sent by fax within 24 hours of site awareness of the death.
- Serious adverse events other than death, regardless of relationship, will be reported via fax or email by the site within 72 hours of becoming aware of the event.

Other supporting documentation of the event may be requested by the review boards and should be provided as soon as possible. All SAEs will be followed until satisfactory resolution or until the Principal Investigator or Subinvestigator deems the event to be chronic or the patient to be stable.

ICH GCP 6, Section 4.11 require that an investigator notifies the sponsor, regulatory authority(ies) and the local IRB immediately of any serious adverse event, deaths, or life-threatening problems that occur in the study.

9.3.2 Type and Duration of the Follow-up of Subjects After Adverse Events

Adverse events will be followed by the study team until resolved or considered stable. If by Day 42 the AE has not resolved the patient will be followed after the normal observation period.

9.4 Halting Rules

The study will be at least temporarily halted, pending a review by the IRB of record, if there are more than 5 serious adverse events classified as related to the study medication in the study.

This study or part of this study may be terminated or suspended at any time. If a study or part of a study is terminated or suspended, the investigators will promptly inform the institutions and the regulatory authorities. The IRBs should be promptly informed and provided the reason(s) for the termination or suspension by the investigator/institution, as specified by the applicable regulatory requirement(s).

10 STATISTICAL CONSIDERATIONS

10.1 Study Outcome Measures and Analysis

The primary outcome for the clinical part of the study will be cure rates in arms 1 and 2. Cure rates will be analyzed separately for Day 28 and 42.

The primary outcome of this study is a nominal variable: the treatment will be characterized as successful or unsuccessful/resistant. Cure rates will be summarized along with 95% confidence intervals (exact based on binomial distribution). The resulting categorical frequency data between arm 1, 2 and 3 will be compared in Fisher's Exact/ Chi^2 tests.

The secondary endpoints of this study are continuous variables. Overall group differences between secondary endpoints of parasite clearance time (PCT), gametocyte clearance time (GCT), and fever clearance time (FCT) will be compared using the Mann-Whitney U test. In vitro data will be analyzed by nonlinear regression models.

10.2 Sample Size Considerations

Enrollment target for arms 1 and 2 will be 40 evaluable subjects (those completing study endpoints). Arm 3 will be used as a comparator. Subjects will be enrolled in the 3 groups at a ratio of 2:2:1. The dropout rate is expected to be approximately 25%. Any dropouts (e.g. withdrawals, lost-to-follow-ups etc.) will be replaced by continuing randomization until a total number of 100 evaluable subjects is reached. Therefore 134 persons may be enrolled to achieve 100 evaluable subjects. In case the enrollment target of 100 evaluable subjects cannot be met within the approved study duration an application for extension of the study duration will be filed.

The sample size in arm 3 is lower as results from previous studies are available for historical comparison.

The sample size is calculated based on an expected failure rate (i.e. early and late treatment failures) of 10%. This sample size will allow for an estimation of the prevalence of failures in each of the artesunate arms within 10 percentage points with 95% confidence (WHO 2003, Assessment and monitoring of antimalarial drug efficacy for the treatment of uncomplicated falciparum malaria. Geneva World Health Organization. Document WHO/HTM/RBM/2003.50).

Anticipated population proportion of clinical failures:	10%
Confidence level:	95%
Precision:	10%
Calculated sample size for artesunate arms:	min 35 evaluable subjects

10.3 Participant Enrollment and Follow-Up

134 persons may be enrolled to achieve 100 evaluable subjects. Study participants will be randomly assigned to one of 3 treatment groups at a ratio of 2:2:1 and followed for 42 days.

Historically lost to follow-up in 42 day studies of uncomplicated falciparum malaria has always been low in this area (unpublished data). Patients will be asked to return to the study center for follow-ups. In case patients cannot return for follow-up they will be visited at their homes by study personnel.

11 ACCESS TO SOURCE DATA/DOCUMENTS

The study site will maintain appropriate medical and research records for this trial, in compliance with Section 4.9 of ICH E6 GCP, and regulatory and institutional requirements for the protection of confidentiality of subjects. The database will be kept at Medical University of Vienna. Data will be shared with the investigators. The site will permit authorized representatives and regulatory agencies to examine (and when required by applicable law, to copy) clinical records for the purposes of quality assurance reviews, audits and evaluation of the study safety and progress.

Source data are all information, original records of clinical findings, observations, or other activities in a study necessary for the reconstruction and evaluation of the trial. Examples of these original documents and data records include, but are not limited to, hospital records, clinical and office charts, laboratory notes, memoranda, subjects' diaries or evaluation checklists, pharmacy dispensing records, recorded data from automated instruments, copies or transcriptions certified after verification as being accurate and complete, microfiches, photographic negatives, microfilm or magnetic media, x-rays, and subject files and records kept at the pharmacy, at the laboratories, and medico-technical departments involved in the clinical study.

The study results will be disseminated to policy makers, researchers, and the communities who took part in this study as to allow for a timely and adequate response to a potentially emerging problem of drug resistance.

12 ETHICS/PROTECTION OF HUMAN SUBJECTS

12.1 Declaration of Helsinki

The investigator will ensure that this study is conducted in full conformity with the current revision of the Declaration of Helsinki (Fifth revision October 2000), or with the International Conference for Harmonization Good Clinical Practice (ICH-GCP) regulations and guidelines, whichever affords the greater protection to the subject.

12.2 Institutional Review Board

The protocol and informed consent documents will be provided for the review and approval.

The protocol will require scientific review and approval by the committee at ICDDR,B. The protocol will undergo ethical review and require approval by the Ethical Review Committee (ERC) of the International Centre for Diarrhoeal Disease Research, Bangladesh, Dhaka, Bangladesh (FWA #1468), the Ethical Review Board of the Medical University of Vienna (FWA #3129), and the Research Ethics Review Committee of the World Health Organization.

If modifications are required, they will be submitted in writing to the Ethical Review Committee (ERC) of the International Centre for Diarrhoeal Disease Research, Bangladesh, Dhaka, Bangladesh (FWA #1468) and the Ethical Review Board of the Medical University of Vienna (FWA #3129). A revised consent form will accompany any request for modification that changes any issue addressed in the currently approved consent form.

All amendments involving major changes to the protocol and/or informed consent form must be reviewed and approved by IRB/IEC and/or local authorities before being implemented. These amendments should not be implemented until all necessary approvals have been obtained, except where necessary to eliminate an immediate hazard(s) to study subjects.

12.3 Informed Consent

Freely given informed consent will be obtained from every subject prior to study participation. Informed consent must take place before any study specific procedure, prior to the initiation of non-routine study-related tests, and prior to administration of study drug. Signed and dated, informed consent will be obtained from each subject (or his/her legally acceptable representative) in accordance with GCPs and with local regulatory and legal requirements. The completed informed consent form must be retained by the investigator as part of the study records and a copy will be provided to study subjects. The investigators, or a person designated by the investigators, will fully inform the subject or, if the subject is unable to provide informed consent, the subject's legally acceptable representative, of all pertinent aspects of the trial

including the written information given approval/favorable opinion by the IRB/IEC. Neither the investigator, nor the trial staff, will coerce or unduly influence a subject to participate or to continue to participate in the study.

In obtaining and documenting informed consent, the investigators will comply with the applicable regulatory requirement(s), and will adhere to GCP and to the ethical principles that have their origin in the Declaration of Helsinki. Prior to the beginning of the trial, the investigators will have the IRB/IEC's written approval/favorable opinion of the written informed consent form and any other written information to be provided to subjects.

The written informed consent form and any other written information to be provided to subjects will be revised whenever important new information becomes available that may be relevant to the subject's consent. Any revised written informed consent form, and written information will receive the IRB/IEC's approval/favorable opinion in advance of use. The subject or the subject's legally acceptable representative will be informed in a timely manner if new information becomes available that may be relevant to the subject's willingness to continue participation in the trial. The communication of this information will be documented.

12.3.1 Informed Consent Process

Informed consent is a process that is initiated prior to the individual's agreeing to participate in the study and continuing throughout the individual's study participation. Extensive discussion of risks and possible benefits of participation in this study will be provided to the subjects and their families. Consent forms describing in detail the study procedures and risks are given to the subject and written documentation of informed consent is required prior to enrolling in the study. Consent forms will be IRB approved and the subject will be asked to read and review the document. If the patient cannot read the content of the consent form will be read and explained to him by study personnel. Upon reviewing the document, the study personnel will explain the research study to the subject and answer any questions that may arise. The subjects will sign the informed consent document prior to being enrolled in the study. The subjects should have the opportunity to discuss the study with their surrogates or think about it prior to agreeing to participate. The subjects may withdraw consent at any time throughout the course of the study. A copy of the informed consent document will be given to the subjects for their records. The rights and welfare of the subjects will be protected by emphasizing to them that the quality of their medical care will not be adversely affected if they decline to participate in this study.

12.4 Subject Confidentiality

All personal study subject data collected and processed for the purposes of this study should be managed by the investigators and his/her staff with adequate precautions to ensure the confidentiality of those data, and in accordance with applicable national and/or local laws and regulations on personal data protection.

Monitors, auditors and other authorized agents, the ethics committees approving this research will be granted direct access to the study subjects' original medical records for verification of clinical trial procedures and/or data, without violating the confidentiality of the subjects, to the extent permitted by the law and regulations. In any presentations of the results of this study at meetings or in publications, the subjects' identity will remain confidential.

12.5 Future Use of Stored Specimens

After the study is completed and all protocol related test procedures have been completed residual specimens will be anonymized. Before anonymization samples may only be used for research purposes other than those mentioned in the protocol after notification and approval by the IRB of record. Anonymized samples can be used for tests not explicitly mentioned in the protocol. During the trial specimens will be stored in a safe place at the research laboratory at the study site, which is only accessible to study staff.

Study specimens will be stored for up to 20 years. The samples will be stored at the MARIB field site, the Medical University, or the ICDDR,B. After study closure the samples will only be tested after anonymization.

The principal investigators (Drs. H. Noedl and WA. Khan) are jointly responsible for specimen storage and future use. Any testing of study samples outside of the consortium/institutions mentioned in the protocol will require approval by both principal investigators.

13 LITERATURE REFERENCES

Adjuik M, Agnamey P, Babiker A et al. Amodiaquine-artesunate versus amodiaquine for uncomplicated Plasmodium falciparum malaria in African children: a randomised, multicentre trial. Lancet 2002 Apr 20;359(9315):1365-72.

Breman JG. The ears of the hippopotamus: manifestations, determinants, and estimates of the malaria burden. Am J Trop Med Hyg. 2001 Jan-Feb;64(1-2 Suppl):1-11.

Brewer TG, Peggins JO, Grate SJ et al. Neurotoxicity in animals due to arteether and artemether. Trans R Soc Trop Med Hyg 1994 Jun;88 Suppl 1:S33-6.

Bunnag D, Viravan C, Looareesuwan S et al. Clinical trial of artesunate and artemether on multidrug resistant falciparum malaria in Thailand: a preliminary report. SE Asian J Trop Med Publ Health 1991; 22:380-85.

Bustos MD, Gay F, Diquet B. In-vitro tests on Philippine isolates of Plasmodium falciparum against four standard antimalarials and four qinghaosu derivatives. Bull World Health Organ. 1994;72(5):729-35.

Denis MB, Tsuyuoka R, Poravuth Y, Narann TS, Seila S, Lim C, Incardona S, Lim P, Sem R, Socheat D, Christophel EM, Ringwald P. Surveillance of the efficacy of artesunate and mefloquine combination for the treatment of uncomplicated falciparum malaria in Cambodia. Trop Med Int Health. 2006 Sep;11(9):1360-6.

Denis MB, Tsuyuoka R, Lim P, Lindegardh N, Yi P, Top SN, Socheat D, Fandeur T, Annerberg A, Christophel EM, Ringwald P. Efficacy of artemether-lumefantrine for the treatment of uncomplicated falciparum malaria in northwest Cambodia. Trop Med Int Health. 2006 Dec;11(12):1800-7.

Doherty JF, Sadiq AD, Bayo L, Alloueche A et al. A randomized safety and tolerability trial of artesunate plus sulfadoxine--pyrimethamine versus sulfadoxine-pyrimethamine alone for the treatment of uncomplicated malaria in Gambian children. Trans R Soc Trop Med Hyg. 1999 Sep-Oct;93(5):543-6.

Dondorp A, Nosten F, Stepniewska K, Day N, White N; South East Asian Quinine Artesunate Malaria Trial (SEAQUAMAT) group. Artesunate versus quinine for treatment of severe falciparum malaria: a randomised trial. Lancet. 2005 Aug 27-Sep 2;366(9487):717-25.

Dupont WD and Plummer WD: PS power and sample size program. Controlled Clin Trials,1997;18:274.

Genovese RF, Newman DB, Brewer TG. Behavioral and neural toxicity of the artemisinin antimalarial, arteether, but not artesunate and artelinate, in rats. Pharmacol Biochem Behav 2000 Sep;67(1):37-44.

Gogtay NJ, Kadam VS, Karnad DR, Kanbur A, Kamtekar KD, Kshirsagar NA. Probable resistance to parenteral artemether in Plasmodium falciparum: case reports from Mumbai (Bombay), India. Ann Trop Med Parasitol. 2000 Jul;94(5):519-20.

Haque R, Thriemer K, Wang Z, Sato K, Wagatsuma Y, Salam MA, Akther S, Akter J, Fukuda M, Miller RS, Noedl H. Therapeutic efficacy of artemether-lumefantrine for the treatment of uncomplicated Plasmodium falciparum malaria in Bangladesh. Am J Trop Med Hyg. 2007 Jan;76(1):39-41.

Hien TT, White NJ. Qinghaosu. Lancet 1993:341:603-8

Karbwang J, Na-Bangchang K. Clinical pharmakology of artemisinin compounds. In: Karbwang J, Wernsdorfer WH, eds. Clinical pharmakology of antimalarials. Bangkok: Faculty of Tropical Medicine, 1993: 263-304.

Li GQ, Guo XB, Fu LC et al. Clinical trials of artemisinin and its derivatives in the treatment of malaria in China. Trans Roy Soc Trop Med Hyg 1994; 88: suppl. 1, 5-6.

Li GQ, Fu YX and Bian WX. Comparison on treatment of falciparum malaria with different courses of artesunate tablet (Chinese). Chung Kuo Chung Hsi I Chieh Ho Tsa Chih 1997; 17: 143-4.

Looareesuwan S. Overveiw of clinical studies on artemisinin derivatives in Thailand. Trans Roy Soc Trop Med Hyg 1994; 88: Suppl. 1, 88-90.

Looaresuwan S, Vanijanonta S, Viravan C, et al. Randomised trial of mefloquine-tetracycline and quinine-tetracycline for acute complicated falciparum malaria. Acta Tropica 1994; 57:47-53.

Looareesuwan S, Wilairatana P, Vanijanonta S, Pitisuttithum P, Ratanapong Y, Andrial M. Monotherapy with sodium artesunate for uncomplicated falciparum malaria in Thailand: a comparison of 5- and 7-day regimens. Acta Trop 1997 Sep 30;67(3):197-205.

Luxemburger C, Brockman A, Silamut K, Nosten F, van Vugt M, Gimenez F, Chongsuphajaisiddhi T, White NJ. Two patients with falciparum malaria and poor in vivo responses to artesunate. Trans R Soc Trop Med Hyg. 1998 Nov-Dec;92(6):668-9.

McGready R, Cho T, Cho JJ et al. Artemisinin derivatives in the treatment of falciparum malaria in pregnancy. Trans R Soc Trop Med Hyg 1998 Jul-Aug;92(4):430-3.

McGready R, Cho T, Keo NK et al. Artemisinin antimalarials in pregnancy: a prospective treatment study of 539 episodes of multidrug-resistant Plasmodium falciparum.Clin Infect Dis. 2001 Dec 15;33(12):2009-16.

Meshnick SR. Artemisinin: mechanisms of action, resistance and toxicity. Int J Parasitol. 2002 Dec 4;32(13):1655-60.

Noedl H. Artemisinin resistance: how can we find it? Trends Parasitol. 2005 Jul 18

Noedl H, Attlmayr B, Wernsdorfer WH, Kollaritsch H, Miller RS. A histidine-rich protein 2-based malaria drug sensitivity assay for field use. Am J Trop Med Hyg. 2004 Dec;71(6):711-4.

Noedl H, Wernsdorfer WH, Miller RS, Wongsrichanalai C. Histidine-rich protein II: a novel approach to malaria drug sensitivity testing. Antimicrob Agents Chemother. 2002 Jun;46(6):1658-64.

Noedl H, Wernsdorfer WH, Krudsood S, Wilairatana P, Viriyavejakul P, Kollaritsch H, Wiedermann G, Looareesuwan S. *In vivo-in vitro* model for the assessment of clinically relevant antimalarial cross-resistance. Am J Trop Med Hyg. 2001 Dec;65(6):696-9.

Nontprasert A, Pukrittayakamee S, Dondorp AM, Clemens R, Looareesuwan S, White NJ. Neuropathologic toxicity of artemisinin derivatives in a mouse model. Am J Trop Med Hyg 2002 Oct;67(4):423-9

Pickard AL, Wongsrichanalai C, Purfield A, Kamwendo D, Emery K, Zalewski C, Kawamoto F, Miller RS, Meshnick SR. Resistance to antimalarials in Southeast Asia and genetic polymorphisms in pfmdr1. Antimicrob Agents Chemother. 2003 Aug;47(8):2418-23.

Price R, Van Vugt M, Phaipun et al. Adverse effects in patients with acute falciparum malaria treated with artemesinin derivatives. Am J Trop Med Hyg 1999; 60:547-55.

Purfield A, Nelson A, Laoboonchai A, Congpuong K, McDaniel P, Miller RS, Welch K, Wongsrichanalai C, Meshnick SR. A new method for detection of pfmdr1 mutations in Plasmodium falciparum DNA using real-time PCR. Malar J. 2004 May 7;3:9.

Ringwald P, Bickii J, Basco LK. In vitro activity of dihydroartemisinin against clinical isolates of Plasmodium falciparum in Yaounde, Cameroon. Am J Trop Med Hyg 1999 Aug;61(2):187-92.

Sahr F, Willoughby VR, Gbakima AA, Bockarie MJ. Apparent drug failure following artesunate treatment of Plasmodium falciparum malaria in Freetown, Sierra Leone: four case reports. Ann Trop Med Parasitol. 2001 Jul;95(5):445-9.

Teja-Isavadharm P, Watt G, Eamsila C et al. Comparative pharmacokinetics-effect kinetics of oral artesunate in healthy volunteers and patients with uncomplicated malaria. Am J Trop Med Hyg. 2001 Dec;65(6):717-21.

Thimasarn K, Jatapadma S, Vijaykadga S, Sirichaisinthop J, Wongsrichanalai C. Epidemiology of Malaria in Thailand. J Travel Med. 1995 Jun 1;2(2):59-65.

Vijaykadga S, Rojanawatsirivej C, Cholpol S, Phoungmanee D, Nakavej A, Wongsrichanalai C. In vivo sensitivity monitoring of mefloquine monotherapy and artesunate-mefloquine combinations for the treatment of uncomplicated falciparum malaria in Thailand in 2003. Trop Med Int Health. 2006 Feb;11(2):211-9.

von Seidlein L, Milligan P, Pinder M et al. Efficacy of artesunate plus pyrimethamine-sulphadoxine for uncomplicated malaria in Gambian children: a double-blind, randomised, controlled trial. Lancet 2000 Jan 29;355(9201):352-7.

Wongsrichanalai C, Wimonwattrawatee T, Sookto P et al. In vitro sensitivity of Plasmodium falciparum to artesunate in Thailand. Bull WHO 1999;77:392-8.

Wongsrichanalai C, Wimonwattrawatee T, Sookto P et al. In vitro sensitivity of Plasmodium falciparum to artesunate in Thailand. Bull WHO 1999;77:392-8.

Wongsrichanalai C, Pickard AL, Wernsdorfer WH, Meshnick SR. Epidemiology of drug-resistant malaria. Lancet Infect Dis. 2002 Apr;2(4):209-18.

Wongsrichanalai C, Sirichaisinthop J, Karwacki JJ, Congpuong K, Miller RS, Pang L, Thimasarn K. Drug resistant malaria on the Thai-Myanmar and Thai-Cambodian borders. Southeast Asian J Trop Med Public Health. 2001 Mar;32(1):41-9.

Wongsrichanalai C, Wimonwattrawatee T, Sookto P, Laoboonchai A, Heppner DG, Kyle DE, Wernsdorfer WH. In vitro sensitivity of Plasmodium falciparum to artesunate in Thailand. Bull World Health Organ. 1999;77(5):392-8.

Wongsrichanalai C, Webster HK, Wimonwattrawatee T, Sookto P, Chuanak N, Thimasarn K, Wernsdorfer WH. Emergence of multidrug-resistant Plasmodium falciparum in Thailand: in vitro tracking. Am J Trop Med Hyg. 1992 Jul;47(1):112-6.

World Health Organization. Severe falciparum malaria. Trans R Soc Trop Med Hyg 2000; 94,Suppl 1:1-90.

World Health Organization. Assessment and Monitoring of Antimalarial Drug Efficacy for the Treatment of Uncomplicated Falciparum Malaria. WHO/HTM/RBM/2003.50. Geneva, 2003.

World Health Organization. World Malaria report 2005. Last accessed 24 Feb 2006. http://rbm.who.int/wmr2005/html/a1_1.htm

Appendix A: Schedule of Procedures/Evaluations

Study Assessments Table

	Baseline (Day 0)	Day 1	Day 2	Day 3	Day 4	Day 5	Day 6	Day 7	Day 14	Day 21	Day 28	Day 35	*End of Study* Day 42
Informed Consent	X												
Eligibility Criteria	X												
Demographics	X												
Medical History	X												
Physical Examination*	X	X	X	X	X	X	X	X	X	X	X	X	X
Prior/Concomitant Medication *	X	X	X	X	X	X	X	X	X	X	X	X	X
Vital Signs*	X	X	X	X	X	X	X	X	X	X	X	X	X
Adverse Events*	X	X	X	X	X	X	X	X	X	X	X	X	X
Clinical Signs & Symptoms*	X	X	X	X	X	X	X	X	X	X	X	X	X
Microbiology: *Thick and Thin Blood Smear***	X	X	X	X	X	X	X	X	X	X	X	X	X
Clinical Laboratory: *Urine Pregnancy*	X												
Clinical Laboratory: *Hematology, Blood Chemistry****	X			X									
Drug levels****	X						X						
PCR (DNA) #	X												
In Vitro Culture #	X												
Subject Summary													X
Study Treatment	X	X	X	X	X	X	X						

* Daily for 7 days, or until parasite clearance, whichever is longer; and on Days 7, 14, 21, 28, 35 and 42

** Daily until aparasitemic on at least 2 successive smears; and on Days 7, 14, 21, 28, 35, and 42

*** Or whenever clinically warranted

**** Drug levels on Day 0 and 6

\# To be repeated on day of recrudescence

Appendix B: Definitions

- **Uncomplicated malaria** is defined as falciparum malaria in the absence of any signs of severe malaria.
- **Severe malaria** is defined following WHO criteria (WHO 2000) as falciparum malaria in the presence of any of the following malaria-associated criteria: cerebral malaria, coma or seizures, pulmonary edema, shock, renal failure, jaundice, severe anemia, spontaneous bleeding, hyperparasitemia, hypoglycemia, acidosis, hemoglobinuria, or sustained hyperpyrexia.
- **Adequate Clinical and Parasitological Response (ACPR)**
  • Absence of parasitemia on Day 28 and 42 irrespective of axillary temperature without previously meeting any of the criteria of Early Treatment Failure or Late Clinical Failure or Late Parasitological Failure.
- **Early Treatment Failure (ETF)**
  • Development of danger signs (e.g. impaired conciousness, convulsions, respiratory distress) or severe malaria on Day 1, Day 2 or Day 3, in the presence of parasitemia
  • Parasitemia on Day 2 higher than Day 0 count irrespective of axillary temperature
  • Parasitemia on Day 3 with axillary temperature ≥ 37.5 °C
  • Parasitemia on Day 3 ≥ 25 % of count on Day 0.
- **Late Treatment Failure (LTF)**
  - Late Clinical Failure (LCF)
    • Development of danger signs or severe malaria after Day 3 in the presence of parasitemia, without previously meeting any of the criteria of Early Treatment Failure
    • Presence of parasitemia and axillary temperature ≥ 37.5 °C (or history of fever) on any day from Day 4 to Day 28/42 without previously meeting any of the criteria of Early Treatment Failure
  - Late Parasitological Failure (LPF)
    • Presence of parasitemia on any day from Day 7 to Day 42 and axillary temperature < 37.5 °C, without previously meeting any of the criteria of Early Treatment Failure or Late Clinical Failure
